# The NLP-HSF regulatory module contributes to nitrogen-mediated thermotolerance in rice

**DOI:** 10.1016/j.xplc.2025.101522

**Published:** 2025-09-08

**Authors:** Dong-Jie Zhu, Zi-Sheng Zhang, Tao Qing, Juan Gao, Cheng-Bin Xiang, Jian-Xiang Liu

**Affiliations:** 1 State Key Laboratory of Plant Environmental Resilience, College of Life Sciences, Zhejiang University, Hangzhou 310027, China; 2 School of Life Sciences, University of Science and Technology of China, Hefei 230027, China

Dear Editors,

High-temperature stress poses a significant threat to global crop productivity, particularly in staple crops such as rice (*Oryza sativa)*, wheat (*Triticum aestivum)*, and maize (*Zea mays)*. Models predict that global yields of these crops will decline by 3%–8% for every 1°C increase in mean temperature. Over the past two decades, much progress has been made in elucidating the molecular mechanisms underlying plant responses to extreme temperatures, including signal perception, transduction, and transcriptional regulation (Li et al., 2023). As climate change intensifies, developing heat-resilient crops has become a critical challenge for sustainable agriculture. Nitrogen (N), an essential macronutrient, is fundamental to plant growth, metabolism, and stress responses (Liu et al., 2022b). Beyond its nutritional role, N also acts as a signaling molecule that modulates growth and development (Liu et al., 2022b). NIN-like proteins (NLPs), first identified as regulators of nodulation in legumes, are now recognized as master regulators of nitrate (NO_3_−N) signaling and metabolism (Liu et al., 2017, 2022a). In rice, NLP3 translocates from the cytoplasm to the nucleus in response to NO_3_−N, where it binds NO_3_−N-responsive *cis*-elements in the promoters of genes involved in N uptake and assimilation, thereby orchestrating their expression (Zhang et al., 2022b). Field studies further show that heat stress reduces photosynthesis and nitrogen use efficiency (NUE), whereas supplemental N enhances heat tolerance (Wang et al., 2008). However, the molecular basis of this effect remains unclear.

In this study, we show that the N sensor NLP3 plays a critical role in rice thermotolerance, particularly under low N conditions. NLP3 translocates to the nucleus in response to heat stress, where it modulates the expression of heat shock factors (Hsfs). These findings highlight the interplay between N nutrition and thermotolerance in rice, providing novel insights into the integration of nutrient and stress responses in plants.

To examine the role of N in rice thermotolerance, we grew wild-type ZH11 under different concentrations of mixed N supply (0.02 mM, 0.2 mM, 2 mM, 1:1 NO_3_^−^:NH_4_^+^) and assessed their sensitivity to heat stress. Before treatment, plants showed similar height (Supplemental Figure 1A). After exposure to heat stress (45°C for 3 days) followed by recovery at 29°C for 7 days, plants grown under low N were more sensitive to heat stress (Supplemental Figure 1B), and the survival rates were significantly reduced when N was limited (Figures 1A–1F; Supplemental Figure 1C). These results indicate that adequate N nutrition is essential for enhancing thermotolerance in rice.

**Figure 1:**
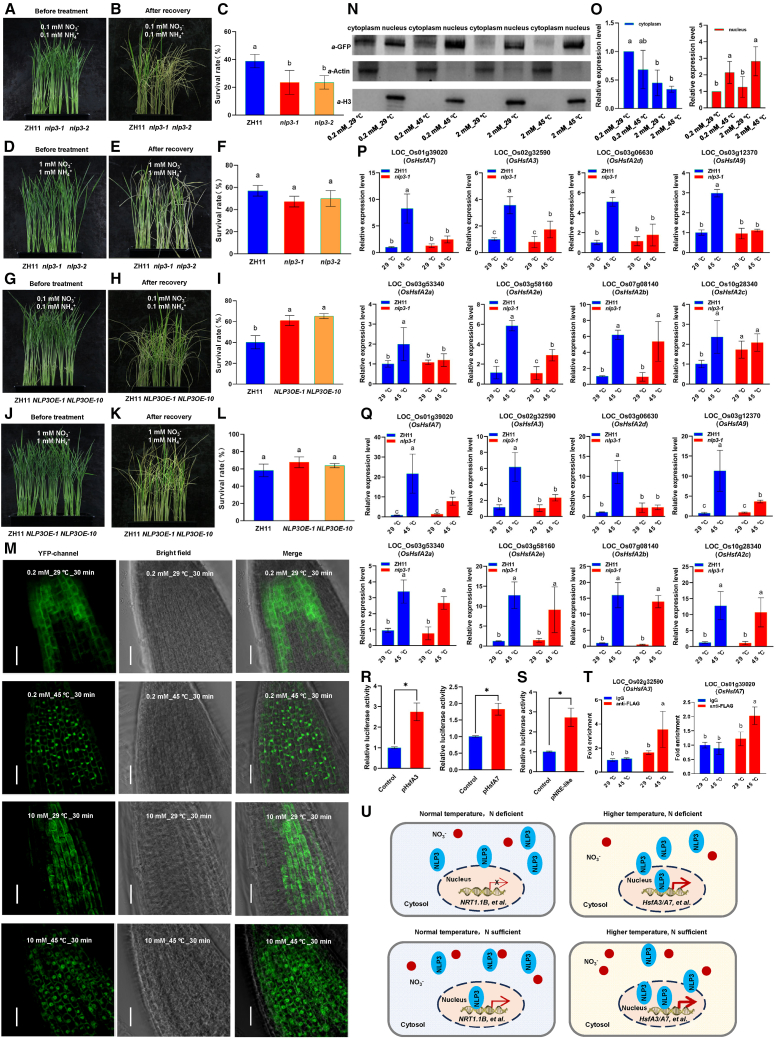
The NLP-HSF module is involved in nitrogen-mediated thermotolerance **(A–L)** Phenotypic analysis. Wild-type ZH11, *nlp3* mutant, and *NLP3* overexpression plants grown under different mixed N conditions at 29°C were subjected to heat stress (45°C, 3 d) and then recovered at 29°C for 7 d. Plants were photographed, and survival rates were calculated. (**M**) Subcellular localization. Seven-day-old *NLP3-GFP* plants pre-cultured under low N (0.2 mM) were treated with high N (10 mM KNO_3_) for 30 min under either normal (29°C) or heat stress (45°C) conditions. Root samples were observed by confocal microscopy. Scale bar, 50 μm. **(N and O)** Cytoplasm-nucleus fractionation. *NLP3-GFP* plants grown under low N (0.2 mM) or normal N (2 mM) were subjected to 45°C for 2 h. Seedlings were fractionated into cytoplasmic and nuclear components, and NLP3-GFP was detected by Western blotting with anti-GFP. Anti-Actin and anti-H3 served as cytoplasmic and nuclear markers, respectively. Band intensities were quantified from three independent blots. **(P and Q)** Gene expression analysis. Plants grown under 0.2 mM **(P)** or 2 mM **(Q)** mixed N conditions at 29°C were subjected to 45°C treatment for 2 h and analyzed by RT-qPCR. **(R and S)** Effector-reporter assays. Promoter sequences of *HsfA3/A7* or the NRE-like motif (TTGACC) were fused to firefly luciferase as reporters, and constitutively expressed NLP3 served as the effector. Renilla luciferase driven by the 35S promoter was used as an internal control. Relative luciferase activity was calculated by normalizing firefly to Renilla activity and then to the empty vector control. **(T)** ChIP-qPCR. Fourteen-day-old *NLP3-FLAG* overexpression plants grown at 29°C under normal N were subjected to 45°C for 2 h and analyzed by ChIP-qPCR. Error bars represent SE (*n* = 3). Asterisks indicate significance compared with the control in *t*-test (∗*p* < 0.05). Different letters denote significant differences based on Tukey’s HSD test (*p* < 0.05). **(U)** Working model for NLP3. Under normal temperatures, NLP3 translocates from the cytoplasm to the nucleus in response to NO_3_−N supply, where it regulates N-responsive genes such as *NRT1*.*1B*. Under heat stress, NLP3 similarly relocates to the nucleus but instead activates a distinct set of heat-responsive genes, including *HsfA3/A7*. N supply promotes nuclear accumulation of NLP3 and enhances heat-responsive gene expression, highlighting the critical role of N nutrition in plant thermotolerance.

To understand how N affects thermotolerance, we performed RNA sequencing (RNA-seq) to compare heat stress responses under N deficiency (LN, 0.2 mM, mixed N supply) and N sufficiency (NN, 2 mM, mixed N supply). We identified 73 genes induced by heat stress under both conditions, with differential expression between N sufficiency and N deficiency at 45°C but not at 29°C (Supplemental Figures 1D and 1E; Supplemental Data 1). GO and KEGG analyses showed significant enrichment of terms including “response to temperature stimuli,” “response to heat,” “protein processing in endoplasmic reticulum,” and “starch and sucrose metabolism” (Supplemental Figure 2). To validate the RNA-seq results, we selected eight *Hsf* genes from this group and performed quantitative reverse-transcription PCR (RT-qPCR). All eight genes were upregulated by heat stress under both N supply conditions, but induction was much stronger under N sufficiency (Supplemental Figure 3). These results indicate that adequate N is required for robust heat stress responses in rice.

The NLP family of transcription factors acts as transcriptional activators that regulate downstream genes involved in N uptake and assimilation. The rice genome encodes six *NLP* genes, and we examined their expression in response to heat stress under different mixed N supply conditions. None of the *NLP* genes were altered by heat stress (Supplemental Figure 4). Because NLP3 responds to N supply and serves as the major NLP regulating NUE and grain yield in rice (Zhang et al., 2022b), we focused on NLP3 in subsequent analyses. We obtained *NLP3* gene-edited mutants (*nlp3-1* and *nlp3-2*; Supplemental Figure 5) and *NLP3* overexpression lines (*NLP3OE-1* and *NLP3OE-10*) (Zhang et al., 2022b) for phenotypic analysis. Under low N conditions (0.2 mM, mixed N supply), the survival rate of *NLP3* mutants was lower than that of ZH11 plants after heat stress and recovery (Figures 1A–1C), whereas the survival rate of *NLP3* overexpression plants was higher than that of ZH11 (Figures 1G–1I). Under N sufficiency (2 mM, mixed N supply), survival rates did not differ among genotypes (Figures 1D–1F and 1J–1L). After heat stress, N content in both shoots and roots was lower in *NLP3* mutants but higher in *NLP3* overexpression plants compared with ZH11 (Supplemental Figure 6), underscoring the importance of N in thermotolerance. ^15^ N-NO_3_^−^ uptake assays further showed that NO_3_−N acquisition was impaired in the *nlp3* mutants in both shoots and roots under heat stress (Supplemental Figure 7). Together, these results demonstrate that NLP3 is required for N-mediated thermotolerance in rice.

NO_3_^−^ and ammonium (NH_4_^+^) are the two primary N sources assimilated by plants. To determine which form of N contributes to thermotolerance in rice, we grew ZH11, *nlp3-1*/*nlp3-2* mutants, and *NLP3OE-1*/*NLP3OE-10* overexpression lines in media supplemented with either NO_3_^−^ or NH_4_^+^ as the sole N source, followed by thermotolerance assays. When grown with 1 mM NO_3_−N, the *nlp3-1*/*nlp3-2* mutants showed significantly greater heat sensitivity than ZH11, whereas the *NLP3OE-1*/*NLP3OE-10* lines displayed enhanced thermotolerance (Supplemental Figures 8A–8D). In contrast, when 1 mM NH_4_^+^ was supplied, no differences in thermotolerance were observed between ZH11 and either the *nlp3* mutants or the *NLP3OE* overexpression lines (Supplemental Figures 8E–8H). These results indicate that NO_3_^−^, rather than NH_4_^+^, plays a critical role in NLP3-mediated thermotolerance in rice.

NLP proteins relocate from the cytoplasm to the nucleus in response to transient N signals (Liu et al., 2017). To examine this process, we developed NLP3-GFP plants and observed the subcellular localization of NLP3-GFP in rice roots under different temperature conditions. Plants grown under N-deficient conditions (0.2 mM, mixed N supply) at 29°C for 7 days were subjected to heat stress (45°C) and N treatment (10 mM) for 30 min. Nuclear GFP signals in roots were more evident under heat stress conditions than under normal temperature (Figure 1M). We also grew NLP3-GFP plants under both N-deficient (0.2 mM, mixed N supply) and N-sufficient (2 mM, mixed N supply) conditions for 7 days and then exposed them to heat stress (45°C) for 2 h. Cytoplasmic and nuclear fractions were isolated from whole seedlings and analyzed by Western blotting. NLP3-GFP accumulated more strongly in the nucleus in response to both N supply and heat stress, with the highest levels under combined N sufficiency and heat stress (Figures 1N and 1O). These results indicate that NLP3 relocates from the cytoplasm to the nucleus under heat stress.

To understand how nucleus-localized NLP3 regulates thermotolerance, we examined the expression of the eight *Hsf* genes mentioned above in ZH11 and *nlp3-1* mutant plants under different N and temperature conditions. Loss of *NLP3* function suppressed the expression of *HsfA7*/A3/A2d/*A9* under both 0.2 mM N and 2 mM N conditions (Figures 1P and 1Q) but did not affect the expression of *NIA1*/*NIA2*/*NRT2*.*4* (Supplemental Figure 9). Expression of *NRT1*.*1B* was upregulated by heat stress in ZH11 plants; however, this induction was impaired in the *nlp3-1* mutant (Supplemental Figure 9). To test whether NLP3 directly regulates *HsfA3/A7/A2d*, we performed effector-reporter assays using their 1.5 kb promoter sequences fused to firefly luciferase (Supplemental Figure 10A). NLP3 activated promoter-driven luciferase activity for *HsfA3/A7/A2d* (Figure 1R; Supplemental Figure 10B), indicating possible direct regulation. Because NLP3 was previously shown to bind to the NO_3_^−^-responsive-like (NRE-like) *cis*-elements, we further tested this using an effector-reporter assay with the TTGACC motif from the *HsfA7* promoter. NLP3 indeed activated this NRE-like *cis*-element (Figure 1S). Chromatin immunoprecipitation (ChIP)-qPCR confirmed that NLP3 directly binds the promoter regions of *HsfA3*/*A7* but not *HsfA2d* under heat stress (Figure 1T; Supplemental Figure 10C). These results demonstrate that NLP3 activates the expression of *HsfA3/A7* under heat stress.

To investigate the functional role of HsfA3 in thermotolerance, we generated two independent mutant lines, *hsfa3-1*/*hsfa3-2* (Supplemental Figures 11A and 11B), and performed phenotypic analysis. These mutants were more sensitive to heat stress than ZH11 plants (Supplemental Figure 12), suggesting that the downstream target gene *HsfA3* is important for thermotolerance in rice.

N is a critical macronutrient for plants, and emerging evidence suggests its involvement in diverse abiotic stress responses, including ionic and drought stress (Liu et al., 2022b). Plants mainly absorb N in the form of NO_3_^−^ and NH_4_^+^. In rice, the high-affinity NO_3_^−^ transporter NRT2.3 exists as two splice variants (NRT2.3a and NRT2.3b) and plays a pivotal role in NUE and yield (Zhang et al., 2022a). Notably, *NRT2*.*3a* expression is negatively regulated by the temperature-dependent small RNA *sNRT2*.*3-1*, whereas rice accessions carrying the *high-temperature-resistant and nitrogen-efficient-2* (*HTNE-2*) allele show enhanced *NRT2*.*3b* translation and improved yield under high nighttime temperatures (Zhang et al., 2022a). These findings establish a direct link between N nutrition and temperature in rice.

In this study, we examined the role of N nutrition in heat stress response and demonstrated that NLP3, a NO_3_^−^ sensor and regulator, integrates N nutrition (especially NO_3_^−^) with thermotolerance in rice by directly regulating *Hsf*s and other heat-responsive genes (Figure 1U). Our findings establish that optimal N nutrition is essential for rice thermotolerance. However, excessive N fertilization can compromise grain quality and contribute to environmental degradation (Liu et al., 2022b). Over the past two decades, major progress in understanding the molecular mechanisms governing NUE in crops, particularly rice, has opened opportunities to enhance thermotolerance through genetic improvement of NUE rather than relying solely on increased fertilizer use. This strategy is particularly promising in high-NUE rice varieties that maintain robust thermotolerance even under low N input conditions.

Rice encodes six *NLP* genes, among which *NLP1*, *NLP3*, and *NLP4* synergistically regulate N utilization (Wu et al., 2021; Zhang et al., 2022b). Our phenotypic analyses show that NLP3, the rice ortholog of *Arabidopsis* NLP7, plays an essential role in thermotolerance, particularly under N-limited conditions. Both *nlp3* mutants and *NLP3*-overexpressing lines exhibited strong thermotolerance phenotypes at 0.2 mM (mixed N) and 1 mM N (pure NO_3_^−^) but not at 2 mM (mixed N), indicating that N status is important for the manifestation of thermotolerance in rice. Notably, *nlp3* mutants displayed impaired heat-responsive gene expression under N sufficiency, and NLP3 protein translocated to the nucleus under heat stress regardless of N availability. These results suggest that while NLP3 contributes significantly to thermotolerance, functional redundancy among NLP family members may compensate for *NLP3* loss under some conditions. Further studies are needed to clarify the cooperative roles of other NLPs in rice thermotolerance.

Heat shock factors (Hsfs) are master regulators of HSPs and other thermotolerance-related genes. The rice genome contains 25 *Hsf* genes, and overexpression of *HSFA2e* confers thermotolerance even in *Arabidopsis* (Guo et al., 2016). We found that NLP3 directly regulates the expression of *HsfA3* and *HsfA7*; however, a previous study reported that *HsfA7* overexpression does not enhance thermotolerance in rice (Liu et al., 2013). Our results show that *nlp3* mutants have significantly reduced induction of key *Hsf*s, including *HsfA2d*, *HsfA3*, *HsfA7*, and *HsfA9*, under heat stress. This suggests that other *Hsf* genes besides *HsfA7* function redundantly downstream of NLP3 to confer thermotolerance. Indeed, we confirmed that HsfA3 is critical for thermotolerance in rice (Supplemental Figure 12). These findings establish NLP3 as a critical regulator of *Hsf* gene expression during the heat stress response. In addition, we observed that N nutrition also modulates the expression of heat-responsive genes beyond the *Hsf* family (Supplemental Data 1), suggesting that NLP3 regulates thermotolerance through both *Hsf*-dependent and *Hsf*-independent pathways. Future transcriptomic comparisons between ZH11 and *nlp3* mutants under varying N and temperature conditions will further clarify the role of NLP3 in thermotolerance.

In summary, our study identifies a previously uncharacterized pathway in which NLP3 mediates thermotolerance by regulating heat-responsive genes, thereby expanding the functional interplay between N nutrition and heat stress adaptation in rice.

## Funding

This project was financially supported by the State Key Project of Research and Development Plan, China (grant no. 2021YFF1000404).

## Acknowledgments

The authors declare no conflict of interest.

## Author contributions

D.-J.Z. and J.-X.L. designed the experiments; D.-J.Z., Z.-S.Z., T.Q., and J.G. performed the experiments; J.-X.L., D.-J.Z., and C.-B.X. analyzed the data; J.-X.L. and D.-J.Z. wrote the paper.

## Supplemental information

Document S1. Supplemental Figures 1–12, Supplemental Table 1, and supplemental materials and methods

Supplemental Data 1. A list of N-dependent heat stress responsive genes

Document S3. Article plus supplemental information
