# Supplementary material for: The NLP-HSF regulatory module contributes to nitrogen-mediated thermotolerance in rice
Source: Plant Commun. 2025 Sep 8;6(11):101522. doi: 10.1016/j.xplc.2025.101522 (PMC12785025; doi:10.1016/j.xplc.2025.101522)
Supplement: Document S1. Supplemental Figures 1–12, Supplemental Table 1, and supplemental materials and methods [file mmc1.pdf]

Plant Communications, Volume *6*

Supplemental information

The NLP-HSF regulatory module contributes to nitrogen-mediated thermotolerance in rice

Dong-Jie Zhu, Zi-Sheng Zhang, Tao Qing, Juan Gao, Cheng-Bin Xiang, and Jian-Xiang Liu

Supplemental information

The NLP-HSF regulatory module contributes to nitrogen-mediated thermotolerance in rice

Dong-Jie Zhu[1], Zi-Sheng Zhang[2], Tao Qing[1], Juan Gao[1], Cheng-Bin Xiang[2] and Jian-Xiang Liu[1, *]

[1]State Key Laboratory of Plant Physiology and Biochemistry, College of Life Sciences, Zhejiang University, Hangzhou 310027, China.

[2] School of Life Sciences, University of Science and Technology of China, Hefei 230027, China.

[*]Correspondence： jianxiangliu@zju.edu.cn.

Supplemental information

Materials and methods

Plant materials and genetic constructs

The wild-type plants used in this study were of the ZH11 background. To generate gene-edited mutants, gene-specific guide sequences (sgRNAs) were designed, and two mutant alleles (*nlp3-1*, *nlp3-2*) were obtained using CRISPR-Cas9 technology (Hangzhou Biogle Co., Ltd, Hangzhou, China). Two *NLP3* overexpression lines (*NLP3OE-1*, *NLP3OE-10*) were generated by cloning the full-length coding sequence (CDS) of *NLP3* into the pCB2006 vector with the constitutive *Actin1* promoter, which was then introduced into ZH11 via *Agrobacterium*-mediated transformation. All primers used are listed in **Table S1**.

Phenotypic analysis under different nitrogen conditions

Seeds were soaked in water at room temperature for 48 hours, followed by germination at 37°C. The germinated seeds were then transferred to black boxes containing modified Kimura B solution. The basic modified Kimura B solution (2 mM) consisted of the following macronutrients: $(NH_4)_2SO_4$ (0.5 mM), KNO_3 (1 mM), $MgSO_4 \cdot 7H_2O$ (0.54 mM), $CaCl_2$ (0.36 mM), K_2SO_4 (0.09 mM), KH_2PO_4 (0.18 mM), and $Na_2SiO_3 \cdot 9H_2O$ (0.7 mM); and micronutrients: $MnCl_2 \cdot 4H_2O$ (9.14 μM), H_3BO_3 (46.2 μM), $Na_2MoO_4 \cdot 2H_2O$ (0.56 μM), $ZnSO_4 \cdot 7H_2O$ (0.76 μM), $CuSO_4 \cdot 5H_2O$ (0.32 μM), and Fe(II)-EDTA (40 μM). For the 0.02 mM and 0.2 mM mixed N treatments, all nitrogen sources were replaced with KNO_3 and NH_4Cl provided as a 1:1 molar ratio of NO_3^- : NH_4^+. For ammonium-only treatment, all nitrogen sources were removed from the modified Kimura B solution and substituted with 1 mM NH_4Cl as the sole nitrogen source. For nitrate-only treatment, all nitrogen sources were eliminated from the modified Kimura B solution and replaced with 1 mM KNO_3 as the exclusive nitrogen source. The nutrient solution was refreshed every two days. The pH was adjusted to 5.5 using HCl.

Growth conditions and heat stress treatment

Plants were cultivated under 20,000 lux light conditions (12-h light/12-h dark cycle) in growth chambers (CONVIRON PGR15). Heat stress was induced by transferring 7-day-old plants from 29°C to a growth chamber set at 45°C for 2–3 days. Subsequently, the plants were returned to 29°C for a 7-day recovery period [1]. Representative plants were photographed, and survival rates were calculated for each experiment.

RNA-seq analysis

For RNA-seq, 7-day-old ZH11 seedlings grown at 29°C under different mixed nitrogen levels (0.2 mM or 2 mM) were subjected to a 2-hour heat treatment (45°C) before sampling. Sequencing was performed on an Illumina HiSeq 4000 platform (LC-Bio Technologies) following standard Illumina protocols [2]. Clean reads were aligned to the reference genome using HISAT2 (v2.2.1; https://daehwankimlab.github.io/hisat2/), and transcript assembly was conducted with StringTie (v2.1.6; http://ccb.jhu.edu/software/stringtie/) using default parameters. Gene expression levels were normalized as fragments per kilobase of transcript per million mapped reads (FPKM). Differentially expressed genes (DEGs) were identified by: DESeq2 (v1.40.2) for pairwise group comparisons. edgeR (v3.42.4) for individual sample comparisons. Genes with $q < 0.05$ and |fold change (FC)| $\geqslant$ 2 were classified as upregulated, while those with $q < 0.05$ and FC $\leqslant$ 0.5 were considered downregulated. GO (Gene Ontology) and KEGG pathway analyses were performed using clusterProfiler (v4.2.2). The top 10 enriched pathways (ranked by P-value) were visualized as bubble plots using ggplot2 (v3.5.1) on the OmicStudio cloud platform (LC-Bio Technology). Statistical significance was assessed using a two-sided test (no multiple-testing correction applied). The RNA-seq data is deposited in the Genome Sequence Archive (GSA) under the accession number (CRA026108).

RT-qPCR analysis

Total RNA was extracted using the RNA Prep Pure Plant Kit (Tiangen, Shanghai,

China) and reverse-transcribed into cDNA with the Evo M-MLV Reverse Transcription Reagent Premix (Accurate Biology, Hangzhou, China). Quantitative real-time PCR (RT-qPCR) was performed using the SuperReal Premix Color kit (Tiangen, Beijing, China) on a CFX96 Real-Time System (Bio-Rad, Hercules, CA, USA). Relative gene expression levels were calculated using the ΔΔCt method. *PP2A* was used as the internal reference gene for normalization. The expression level of the first treatment sample was set to 1, and the relative expression of other samples was calculated accordingly.

^{15}N-nitrate uptake and nitrogen content measurement

^{15}N-uptake assay was used with ^{15}N nitrate-KNO_3 (99 atom % ^{15}N, Macklin, C11510585). For ^{15}N-nitrate uptake assay, 7-day-old wild type (ZH11), mutant (*nlp3*), and OE plants (*NLP3-GFP*) were cultured in the Kimura B solution for 7 days. The plants were pre-treated with the fresh modified Kimura B containing 0.1 or 1 mM ^{15}N-KNO_3 solution for 2 hr, and then transferred from 29°C to 45°C for 2 d. The roots were washed for 1 min in 0.1 mM $CaSO_4$.Then the samples were analyzed for nitrogen content measurement. Samples were collected, dried in an oven, and ground into fine powder using a grinding mill (120 s). Approximately 1 mg of each powdered sample was weighed and analyzed for nitrogen content using an isotope ratio mass spectrometer (IRMS; Thermo Finnigan MAT DELTA plus XP).

Subcellular localization assays

Seven-day-old seedlings of *NLP3-GFP* overexpression plants, pre-cultured in modified Kimura B solution containing 0.2 mM nitrogen, were subjected to high nitrogen (10 mM) KNO_3 treatment for 30 min under either normal (29°C) or heat stress (45°C) conditions. After treatment, root samples were immediately collected for confocal imaging using a two-photon laser scanning microscope (LSM710nlo, Carl Zeiss, Oberkochen, Germany) to visualize NLP3-GFP subcellular localization.

Subcellular protein fractionation assays

Nuclear-cytoplasmic fractionation was carried out following previous published

paper [3]. Seven-day-old seedlings of *NLP3-GFP* overexpression plants grown under low N (0.2 mM) and normal N (2 mM) conditions were subjected to 45°C heat stress for 2 hours and then ground into fine powder in liquid nitrogen using a mortar and pestle. The powder was homogenized in Buffer A containing: 5% Triton X-100, 0.25 M sucrose, 10 mM Tris-HCl (pH 7.8), 10 mM $MgCl_2$, 10 mM KCl, 5 mM β-mercaptoethanol, 1 mM PMSF, 1× Roche protease inhibitor cocktail. The homogenate was filtered through Miracloth (Calbiochem) and incubated on ice for 15 min, and centrifuged at 2,000 × g, 4°C, 5 min. The supernatant was collected as the total protein fraction. For cytosolic protein extraction, the supernatant was further centrifuged at 12,000 × g, 4°C, 10 min. The resulting supernatant represented the cytosolic fraction. The pellet from was resuspended in Buffer A, gently pipetted to mix, and centrifuged at 2,000 × g, 4°C, 10 min. This washing step was repeated twice, discarding the supernatant each time. For nuclear protein extraction, the washed pellet was resuspended in Buffer B containing: 25% glycerol, 0.5 mM DTT, 20 mM Tris-HCl (pH 7.5), 2 mM $MgCl_2$, 400 mM NaCl, 0.2 mM EDTA, 1 mM PMSF, 1× Roche protease inhibitor cocktail. The mixture was incubated on ice for 30 min with gentle mixing and centrifuged at 12,000 × g, 4°C, 15 min. The supernatant was collected as the nuclear protein fraction.

Effector-reporter assay

Effector-reporter assays were performed following previous protocol [4]. Briefly, full-length of *NLP3* (CDS) was cloned into pGreenII 62-SK vector to generate the effector, and 1.5 kb promoter sequence of HsfA7 or 4× tandem repeats of NRE-like motif (TTGACC) was inserted into pGreen0800-II vector to make the firefly luciferase reporter, in which Renilla luciferase driven by 35S promoter was used as an internal control. Different combination of vectors was infiltrated into *N. benthamiana* leaves via *Agrobacterium tumefaciens* (GV3101 strain). After 48-72 hours, luciferase was measured with Dual-Luciferase® Reporter Assay System (Promega, Cat.No. E1910).

ChIP–qPCR

For ChIP-qPCR analysis, 7-day-old *NLP3-FLAG* overexpression plants grown at 29ºC under N normal conditions were subjected to heat stress (45ºC) for 2 hr, and then were harvested for fixation with 1% [w/v] formaldehyde for 15 min, which was stopped by adding 0.125 M glycine for 10 min. Nuclei were then extracted and resuspended in a nuclei lysis buffer. After ultrasonication in 0.8% [w/v] SDS buffer, the mix was immunoprecipitated with *anti*-FLAG (Abmart) or the IgG control. The enrichment of DNA fragments was then quantified by qPCR with routine procedures.

[1] Yang C, Luo A, Lu H-P, et al. Diurnal regulation of alternative splicing associated with thermotolerance in rice by two glycine-rich rna-binding proteins. Science Bulletin, 2024, 69: 59-71

[2] Lu HP, Liu XH, Wang MJ, et al. The NAT1-bHLH110-CER1/CER1L module regulates heat stress tolerance in rice. Nature Genetics, 2025, 57:

[3] Kinkema M, Fan WH, Dong XN. Nuclear localization of NPR1 is required for activation of *PR* gene expression. Plant Cell, 2000, 12: 2339-2350

[4] Liu XH, Lyu YS, Yang W, et al. A membrane-associated nac transcription factor OsNTL3 is involved in thermotolerance in rice. Plant Biotechnology Journal, 2020, 18: 1317-1329

Table S1. Primers used in this study

Name	Purpose	Forward Primer (5'-3')	Reverse Primer (5'-3')
NLP3- target 1	sgRNA	GTTATTCCGGTCCACTACCC	CTCGTCGAACAGCCACAG
NLP3- target 2	sgRNA	CTGCTGTTCTCGTCGGTGT	TGCCCCTCTGCTTCAACTTT
pCB2006- NLP3	Overexpression	ATGGAGGTTGACCCATCGTC	TCAACCTGAGCTTCCACAGGAAC
pCB2006- NLP3:GFP	Overexpression	ATGGAGGTTGACCCATCGTC	ACCTGAGCTTCCACAGGAAC
qLOC_Os01g39020	RT-qPCR	GCTTCTTCAAGCACGCCAAC	AGTGACCTTTCGGAACCCGTAG
qLOC_Os01g62290	RT-qPCR	AACACCGTCTTCGATGCCAAGC	GCACCACAATCATAGGCTTATCGC
qLOC_Os02g02410	RT-qPCR	GTTTGAGGTGTTGGCCACCAATG	TCTCTGGTCAAAGTCCTCACCTC
qLOC_Os02g32590	RT-qPCR	GGACCTCAATCTTGCCATGCTG	TGTCGTCTGGTGCTTCGATTCC
qLOC_Os02g52150	RT-qPCR	GTGGTCAAATTCAGGCGGACTC	TAATTGTCTCGCGCACGGTTC
qLOC_Os03g06630	RT-qPCR	GCCAGCTCAACACCTACTTCTTGG	GGCCTCTCAGGAAACCATCATTCG
qLOC_Os03g12370	RT-qPCR	TTACCGAAGCTTGATTGGTGGTC	AGGTTGGTCACCGAAGAACTAATG
qLOC_Os03g53340	RT-qPCR	TCGCCATTCTCACCGACATGAATC	AAGTCGAGCTCCTCCTCTTTCACG
qLOC_Os03g58160	RT-qPCR	TGTGGGATCCTCACCTCTTTGG	TCCACCTTCCTGAAGCCATAGG
qLOC_Os07g08140	RT-qPCR	AGGCAGCTCAACACCTATGGTTTC	TTGCTGGTTTGAAGCAGTATGGG
qLOC_Os10g28340	RT-qPCR	CTCAACACCTACGGCTTCAGAAAG	TGTTGTGACGGAGGTGCATTGG
qPP2A	RT-qPCR	TTATGGGGGATTATGTGGATCG	GTGCTGTCAGTGGAAAATAGTC
qLOC_Os01g13540	RT-qPCR	GCTGGTGAGGTTGATAAAGTCTGC	GCGGCCTTCATTGCATATCTGG
qLOC_Os01g14420	RT-qPCR	ACGCTGAAAGAGAGAAAGCCAAG	CTTGGAAAGCGCCTGGTGATAC
qLOC_Os03g03900	RT-qPCR	CGCAGAAGGTCAACTACAGTGC	GAGCTCCTGAGATCAACTTCCTTG
qLOC_Os04g41850	RT-qPCR	GAGTTGCTTGCCAACTGGACAC	TGGAACTAGGCTGTACCTGCTC
qLOC_Os09g37710	RT-qPCR	AACTTCACCTCCGAGCTCAATACC	GCTTGTCAGGTTAACTGCCTGGAG
qLOC_Os11g16290	RT-qPCR	TCAAGTGGTGGGTCTTGCTGTG	TGCTTAACTGAACAGACTGCAGAG
qLOC_Os08g36480	RT-qPCR	CCAATTCTTTCATCGTGTTCT	CATGCAGCATTTCGTTTCT
qLOC_Os08g36500	RT-qPCR	ACTGGTGCTGGTGCTTCTGG	CGGCTGGGTGTTGAGGGACT
qLOC_Os10g40600	RT-qPCR	GGCAGGCTCGACTACTTCTA	AGGCGCTTCTCCTTGTAGAC
qLOC_Os01g36720	RT-qPCR	TTCGTCGCGCTCCGGTTCG	CGCACGGGAGTAGGTAGGTG
35S-NLP3	Effector	TCCCCGCGGATGGAGGTTGACCCATCGTC	CGGGATCCTCAACCTGAGCTTCCACAGG
pNRE-like	Reporter	TCGACATTGACCTTGACCTTGACCTTGACC	AGCTGGTCAAGGTCAAGGTCAAGGTCAATG
pHSFA7	Reporter	GCGTCGACAGTGAGGCTCTTGTAACC	CCAAGCTTCGGTGGCAATGCATGGCT
pHSFA3	Reporter	GCGTCGACACCGATCGAGCCAAGCCA	CCAAGCTTGATATGGATTGCGGGGGG
pHSFA2d	Reporter	GCGTCGACGAGTGTTTATATACAATAA	CCAAGCTTAATATAAACAACCGTGTA
q-pHSFA7	ChIP-qPCR	CCCTCCGTCCACAGTTTTAC	CCGTGTCCTACGTTTGATCG
q-pHSFA3	ChIP-qPCR	TTCTGAACCTCTATTTTGGC	TAACATCAATATGAATGTGG
q-pHSFA2d	ChIP-qPCR	ATATCGTGTCTGGTAGAATC	CGGTGATTGGCACACACGA

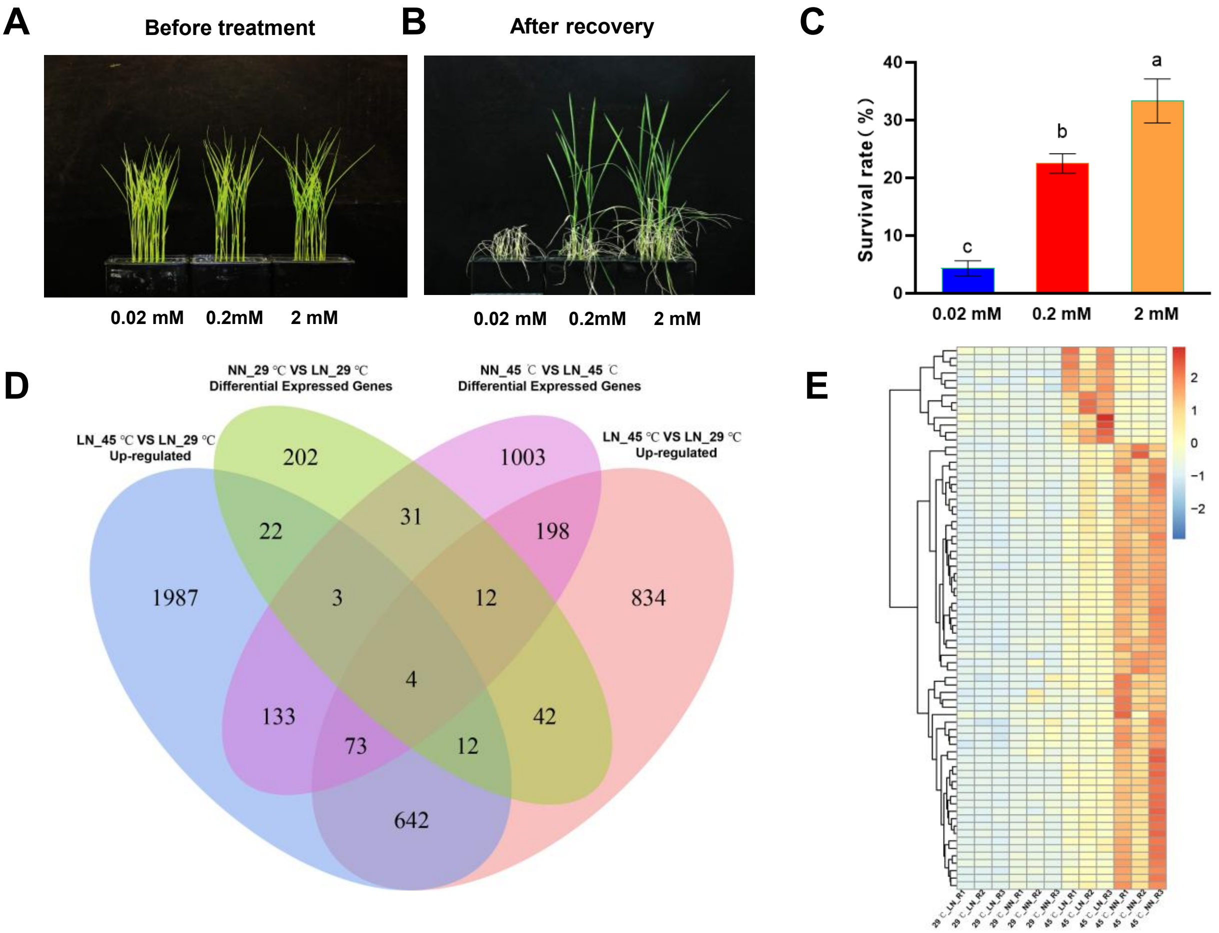

Figure S1. Enhancing heat stress tolerance by nitrogen nutrition. A-C, Phenotypic analysis. Wild-type ZH11 plants cultivated in different nitrogen concentration (1:1 NO_3^-:NH_4^+) at 29°C were subjected to heat stress (45°C) treatment for 3 d and then recovered at 29°C for 7 d, and plants were photographed (A-B) and survival rate (C) was calculated. **D-E**, Transcriptional analysis. ZH11 plants grown under 0.2 mM (LN) or 2 mM (NN) nitrogen conditions at 29°C were subjected to heat stress (45°C) for 2 hr and harvested for RNA-seq analysis (D). The expression of 73 genes involved in nitrogen-mediated heat responses is shown with heat map (E). Error bars represent SE (n = 3). Different letters indicate significant differences as determined by a Tukey's HSD test ($P < 0.05$).

A

B

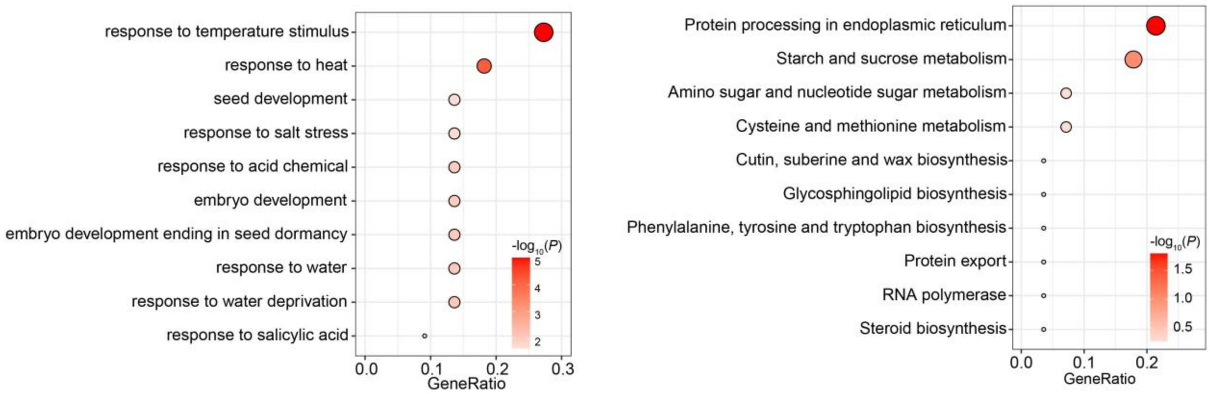

Figure S2. GO and KEGG analysis of N-dependent heat responsive genes. Wild-type ZH11 plants grown under 0.2 mM and 2 mM nitrogen conditions at 29°C were subjected to heat stress (45°C) for 2 hr and harvested for RNA-seq analysis. Totally 73 heat responsive genes related to N supply were used for GO (A) KEGG (B) analysis.

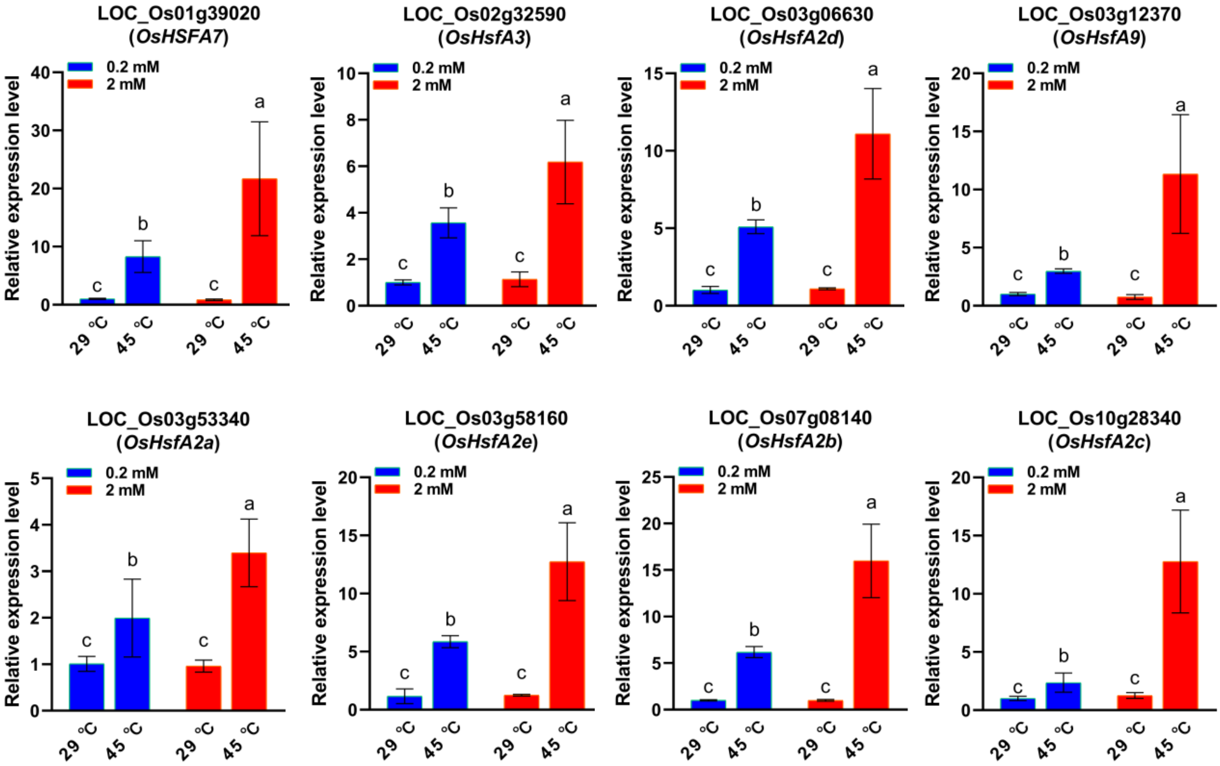

Figure S3. Validation of RNA-Seq analysis. Wild-type ZH11 plants grown under different nitrogen conditions at 29°C were subjected to heat stress (45°C) for 2 hr and harvested for RT-qPCR. Error bars represent SE (n = 3). Different letters indicate significant differences as determined by a Tukey's HSD test ($P < 0.05$).

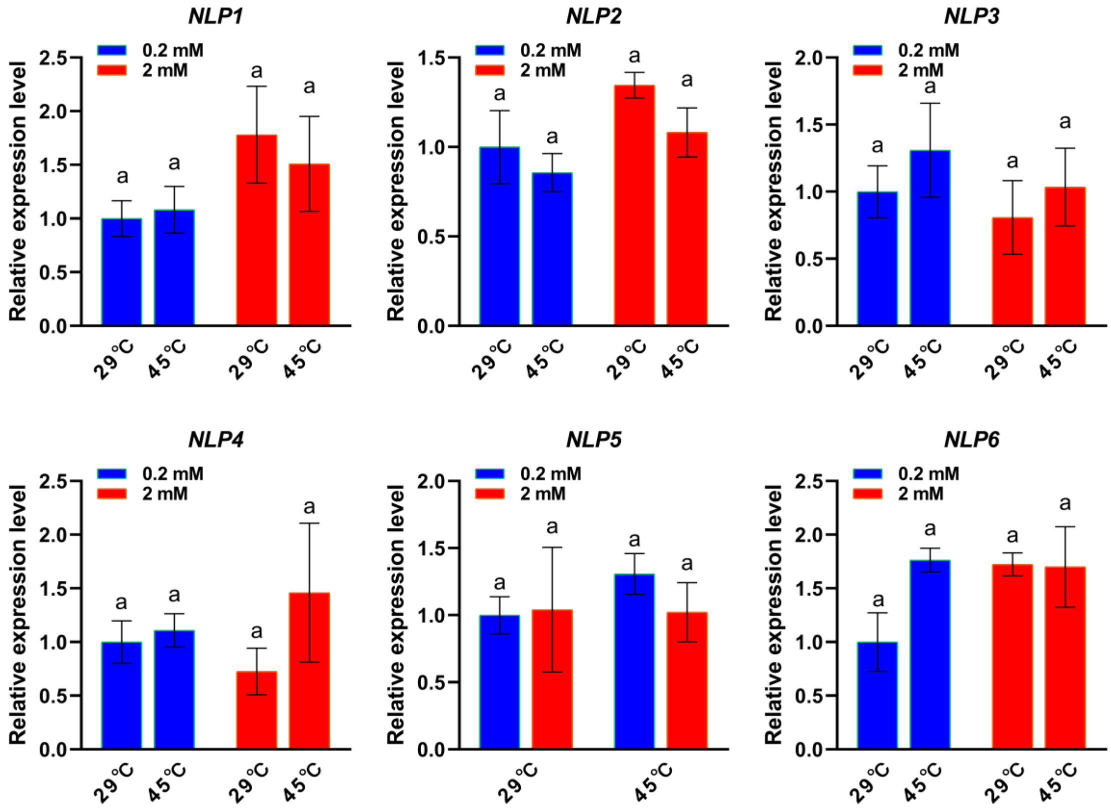

Figure S4. Responses of *NLP1-6* genes to heat stress. ZH11 plants grown under 0.2 mM and 2 mM nitrogen conditions at 29°C were subjected to heat stress (45°C) for 2 hr and harvested for RNA-seq analysis. Error bars represent SE (n = 3). Different letters indicate significant differences as determined by a Tukey's HSD test ($P < 0.05$).

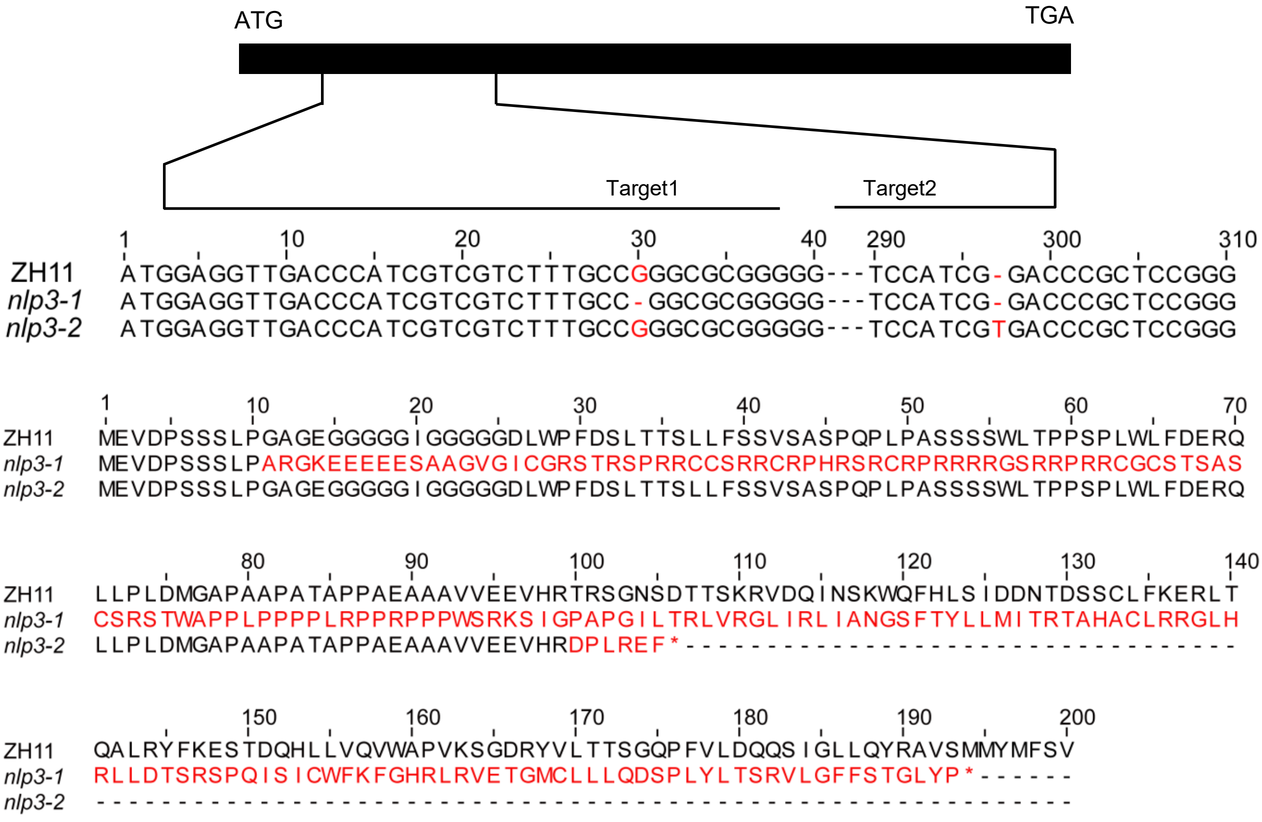

Figure S5. Characterization of *nlp3* mutants. The mutated nucleic acids and protein sequences of NLP3 in wild-type ZH11 and gene-edited mutants (*nlp3-1/-2*).

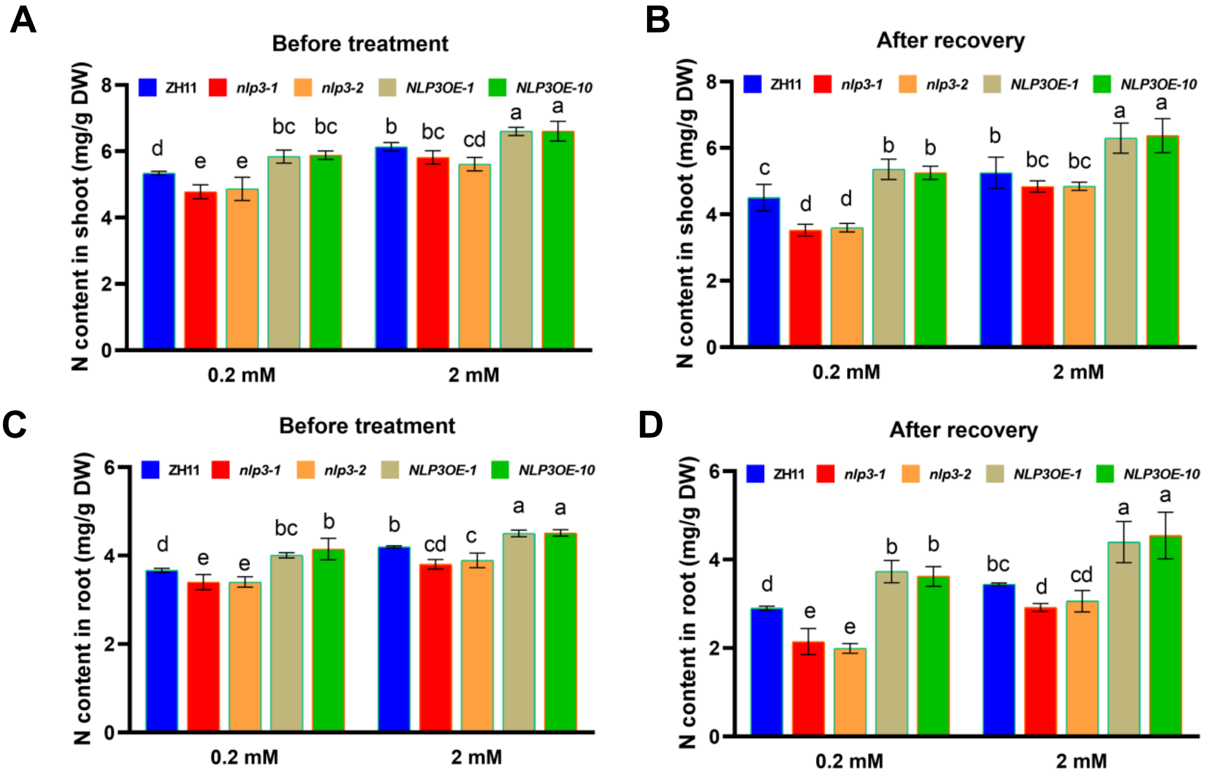

Figure S6. Nitrogen content in *nlp3* mutants and *NLP3* overexpression plants. Wild-type (ZH11), *nlp3* mutant (*nlp3-1/-2*), and *NLP3* overexpression (*NLP3OE-1/-10*) plants grown under 0.2 mM and 2 mM nitrogen conditions at 29°C were subjected to heat stress (45°C) treatment for 3 d and then recovered at 29°C for 7 d. Shoots and roots collected before stress treatment or after 7 d recovery were grounded into fine powder to measure total nitrogen content. Error bars represent SE (n = 3). Different letters indicate significant differences as determined by a Tukey's HSD test ($P < 0.05$).

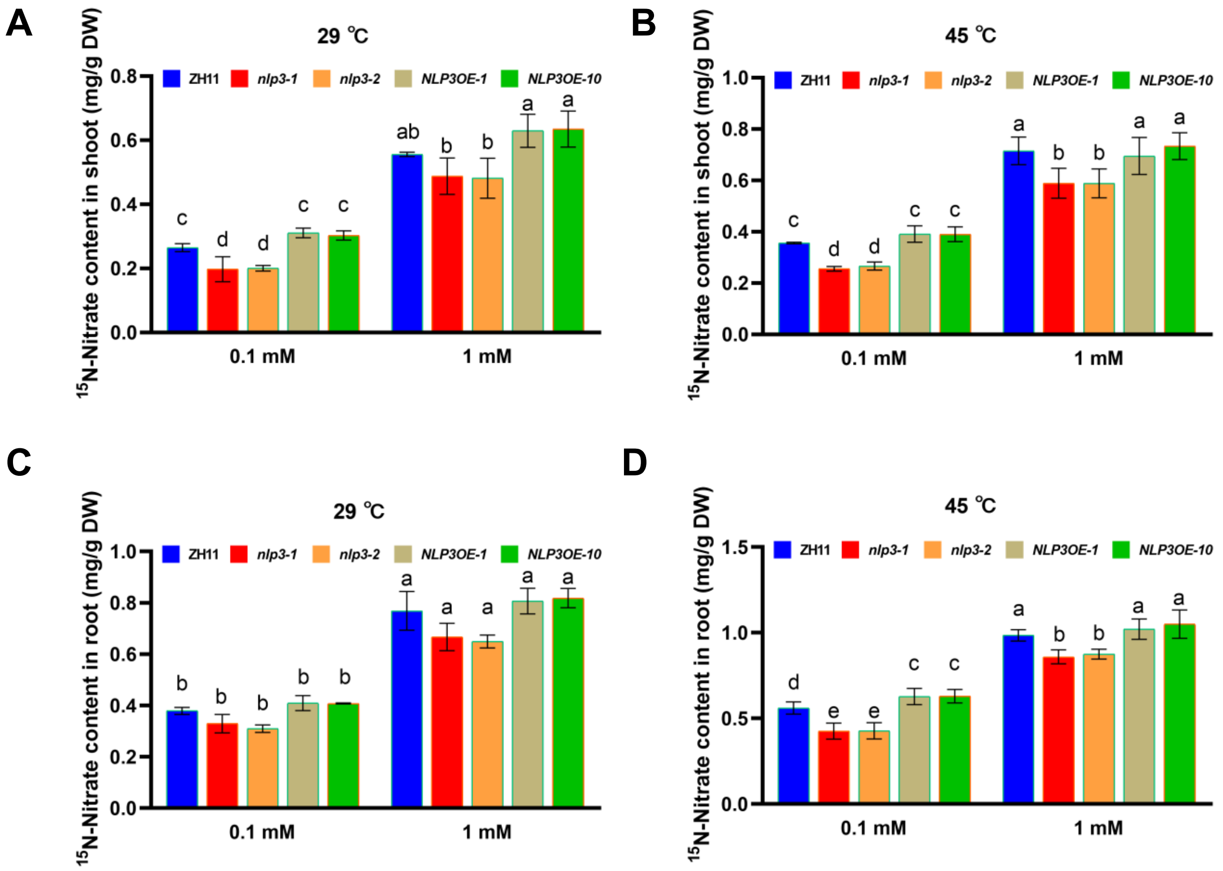

Figure S7. Nitrogen uptake assay with ^{15}N-nitrate. Seven-day old wild-type (ZH11), *nlp3* mutant (*nlp3-1/-2*), and *NLP3* overexpression (*NLP3OE-1/-10*) plants grown under 0.1 mM and 1 mM ^{15}N-KNO3 solution were transferred from 29℃ to 45℃ (heat stress) for 2 d. Shoots and roots were collected to measure ^{15}N-nitrate content. Error bars represent SE (n = 3). Different letters indicate significant differences as determined by a Tukey's HSD test ($P < 0.05$).

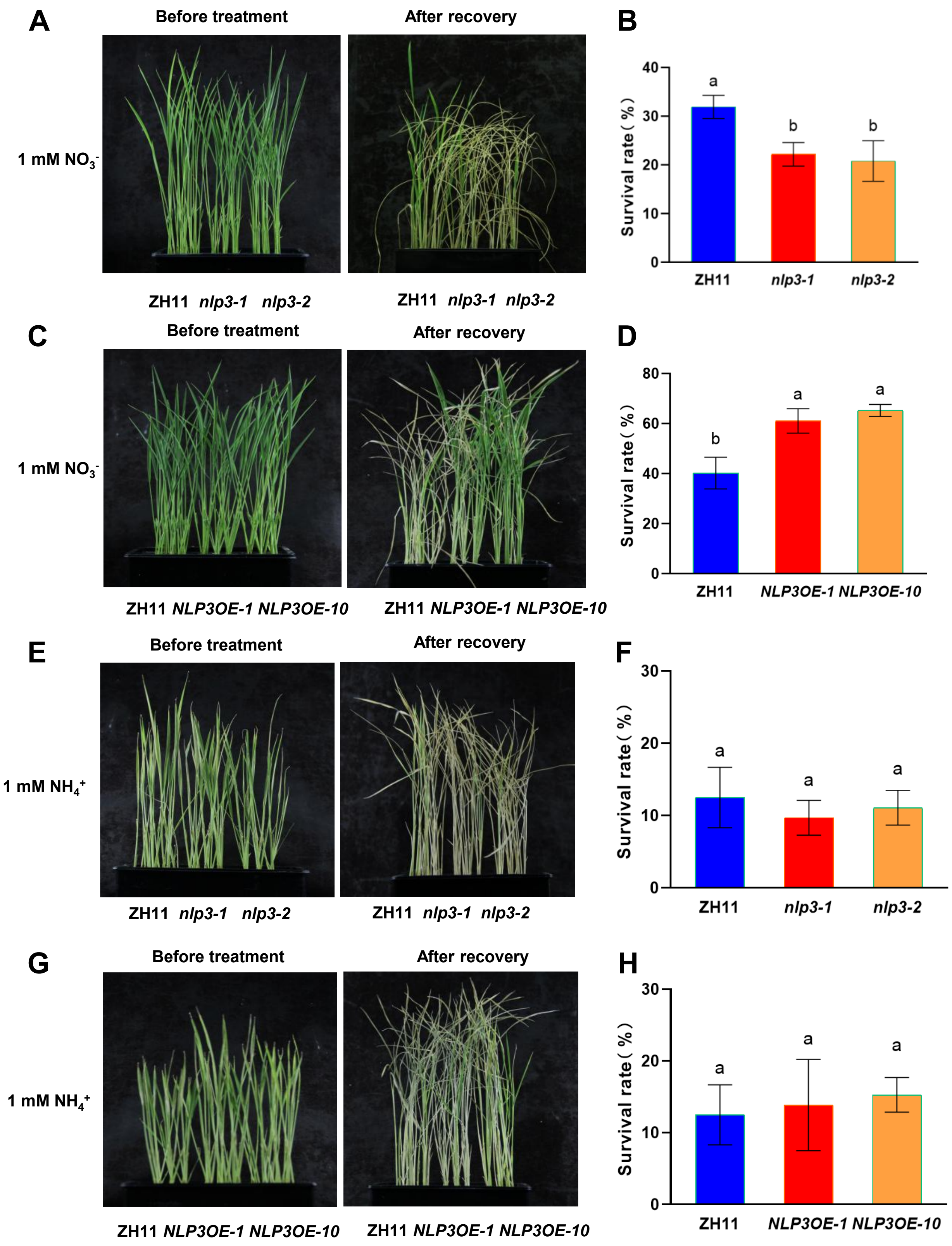

Figure S8. Phenotypic analysis under pure nitrate or pure ammonium conditions. Wild-type (ZH11), *nlp3* mutant (*nlp3-1* and *nlp3-2*), and *NLP3* overexpression (*NLP3OE-1* and *NLP3-10*) plants grown under pure nitrate (A-D) or pure ammonium (E-H) conditions at 29°C were subjected to heat stress (45°C) treatment for 3 d and then recovered at 29°C for 7 d, and plants were photographed and survival rate was calculated. Error bars represent SE (n = 3). Different letters indicate significant differences as determined by a Tukey's HSD test ($P < 0.05$).

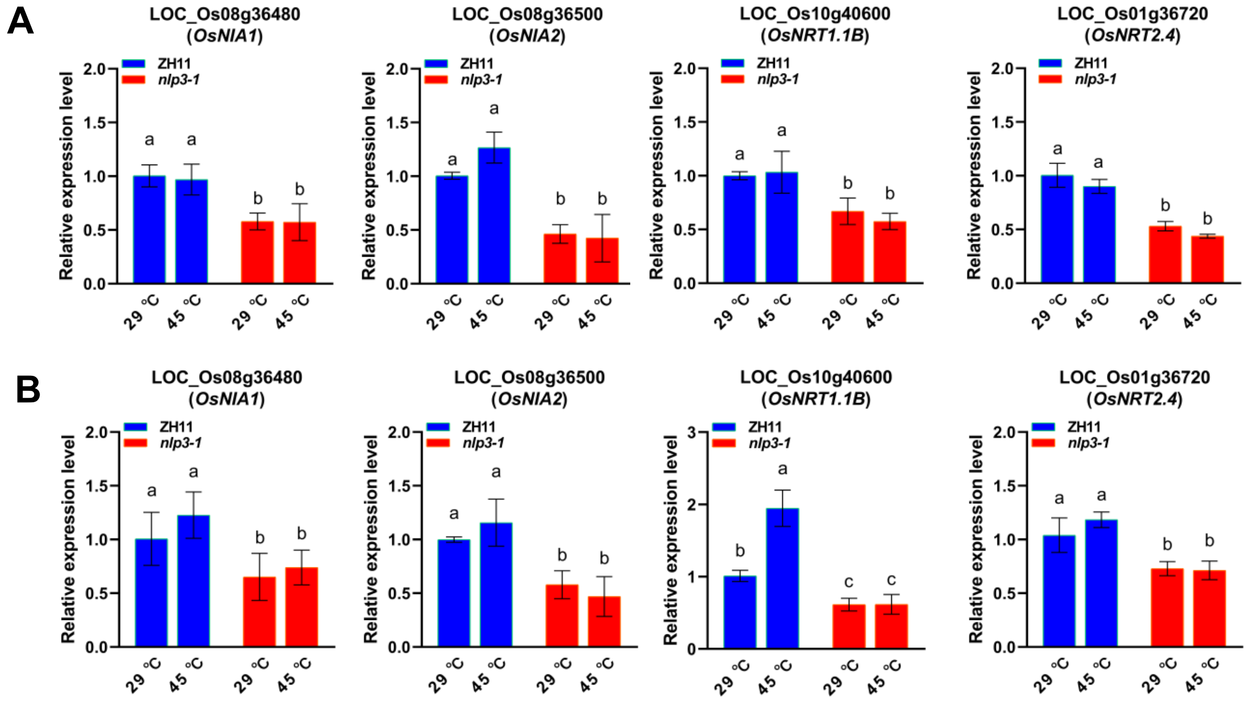

Figure S9. Gene expression analysis of nitrogen metabolism genes. Plants grown under 0.2 mM (A) and 2 mM (B) mixed nitrogen conditions at 29°C were subjected to 45°C treatment for 2 hr and harvested for RT-qPCR analysis. Error bars represent SE (n = 3). Different letters indicate significant differences as determined by a Tukey's HSD test ($P < 0.05$).

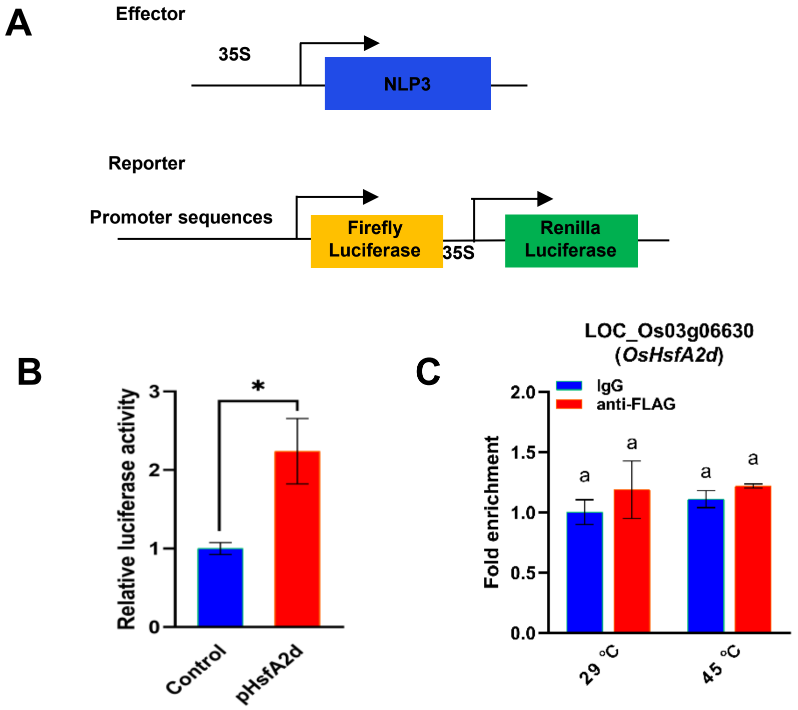

Figure S10. NLP3 does not directly control the expression of HsfA2d. *HsfA3/A7/A2d* promoter sequence or NRE-like motif (TTGACC) is linked with firefly luciferase to make the reporter while constitutively expressed NLP3 is an effector, in which Renilla luciferase driven by 35S promoter is used as an internal control (A). The relative luciferase activity is the firefly luciferase activity normalized to the Renilla luciferase activity which was then normalized to the empty vector control (B). 14-day-old *NLP3-FLAG* overexpression plants grown at 30ºC under N normal conditions were subjected to heat stress (45ºC) for 2 hr, and then harvested for ChIP-qPCR (C). Error bars represent SE (n = 3). Asterisks indicate significance levels when comparing to the control in *t*-test. (*, $P < 0.05$). Different letters indicate significant differences as determined by a Tukey's HSD test ($P < 0.05$).

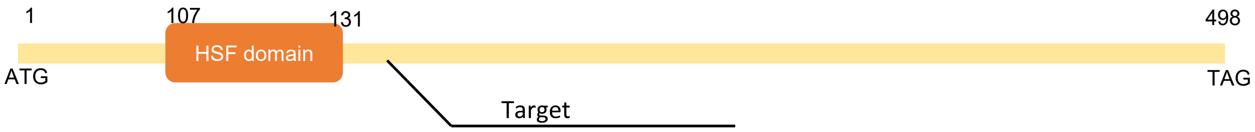


```
ZH11 (WT)     5'-CATGAGGATTTCCTACGACATAGCAAACATTTGTTGAAGAAGATTGTCCGG-CGCAGGTCCTCACCAACACAACAAAGTGGTCTTCAGCCTGGCTCT-3'
hsfa3-1 (+T)  5'-CATGAGGATTTCCTACGACATAGCAAACATTTGTTGAAGAAGATTGTCCGGTCGCAGGTCCTCACCAACACAACAAAGTGGTCTTCAGCCTGGCTCT-3'
hsfa3-2 (+G)  5'-CATGAGGATTTCCTACGACATAGCAAACATTTGTTGAAGAAGATTGTCCGGGCGCAGGTCCTCACCAACACAACAAAGTGGTCTTCAGCCTGGCTCT-3'
```

```
                 10        20        30        40        50        60        70        80        90       100
         ....|....|....|....|....|....|....|....|....|....|....|....|....|....|....|....|....|....|....|....|
ZH1      MDHNTDPPPTTMVDAAAALLLEPKLEGYDDDGGGEPLQPAPFVSPLDQLMQPPRPLEALLQGPQLPPFLSKTYDLVCEPELDGVISWGHAGNSFVVWDPS
hsfa3-1  MDHNTDPPPTTMVDAAAALLLEPKLEGYDDDGGGEPLQPAPFVSPLDQLMQPPRPLEALLQGPQLPPFLSKTYDLVCEPELDGVISWGHAGNSFVVWDPS
hsfa3-2  MDHNTDPPPTTMVDAAAALLLEPKLEGYDDDGGGEPLQPAPFVSPLDQLMQPPRPLEALLQGPQLPPFLSKTYDLVCEPELDGVISWGHAGNSFVVWDPS

                110       120       130       140       150       160       170       180       190       200
         ....|....|....|....|....|....|....|....|....|....|....|....|....|....|....|....|....|....|....|....|
Zh11     AFARDVLPHHFKHNNFSSFVRQLNTYGFRKVHADRWEFAHEDFLRHSKHLLKKIVRRRSSPTQQSGLQPGSSGESGLDPELNTLRREKSALLQEVTRLKQ
hsfa3-1  AFARDVLPHHFKHNNFSSFVRQLNTYGFRKVHADRWEFAHEDFLRHSKHLLKKIVRSQVLTNTTKWSSAWLFW*
hsfa3-2  AFARDVLPHHFKHNNFSSFVRQLNTYGFRKVHADRWEFAHEDFLRHSKHLLKKIVRAQVLTNTTKWSSAWLFW*

                210       220       230       240       250       260       270       280       290       300
         ....|....|....|....|....|....|....|....|....|....|....|....|....|....|....|....|....|....|....|....|
ZH11     EHLQTIEQMSTLNQRLESAEDRQKQMVSFLAKLLQNPTFLRQLKMHRQQKEIDSTRVKRKFLKHVPHGNIDSGESSSQHTGESNLDFSPTSLDLPATHSD
hsfa3-1
hsfa3-2

                310       320       330       340       350       360       370       380       390       400
         ....|....|....|....|....|....|....|....|....|....|....|....|....|....|....|....|....|....|....|....|
ZH11     ILDLQNFLLEDGDLNLAMLPENIGLDGIEAPDDIGALVQGFDTQEELELGSGVELLEIPPASGPRGQDPTIGRSKGKNVLSPGLDATSSEADCLGSFSDN
hsfa3-1
hsfa3-2

                410       420       430       440       450       460       470       480       490       500
         ....|....|....|....|....|....|....|....|....|....|....|....|....|....|....|....|....|....|....|....|
ZH11     MGMLSDSMLQTAGKLMDADDDERIWGVDASSALQSSCSGTSQQAYGSLVSDPYLMEMANKPEKFWELDFQALDDGDLQLDKCVIDDPALQQQRGNMNS*
hsfa3-1
hsfa3-2
```

Figure S11. Characterization of *hsfa3* mutants. The mutated nucleic acids and protein sequences of HsfA3 in wild-type ZH11 and gene-edited mutants (*hsfa3-1/-2*).

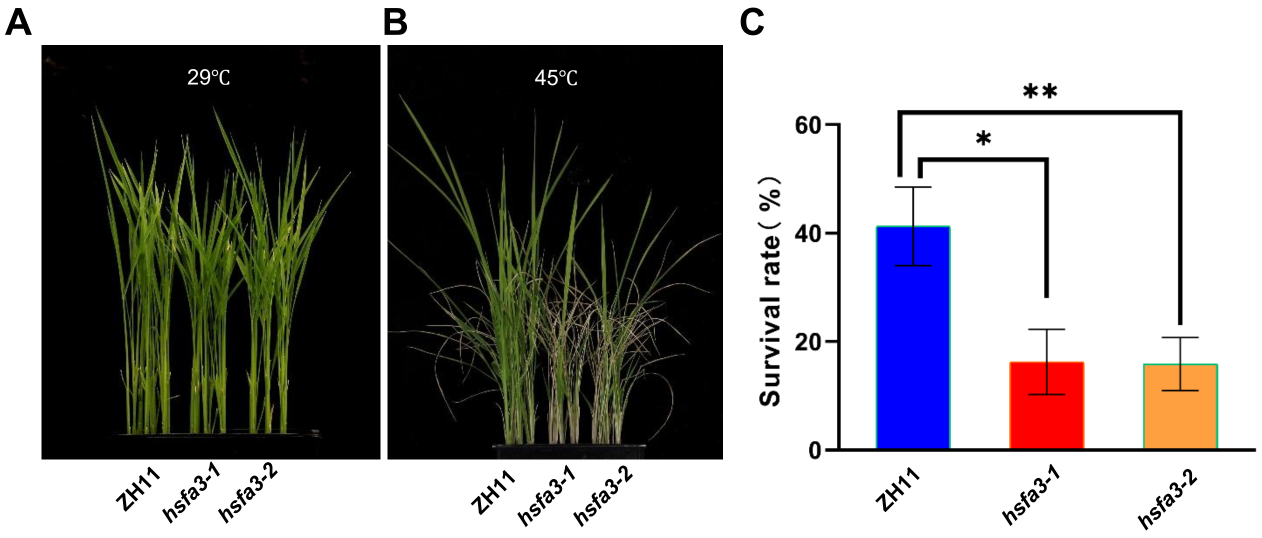

Figure S12. Phenotypic analysis of the *hsfa3* mutants. Seven-day-old wild-type (ZH11), mutants of *HsfA3* (*hsfa3-1/hsfa3-2*) grown at 29°C were subjected to heat stress (45°C) for 2 d and then recovered at 29°C for 14 d, and plants were photographed (A-B) and survival rate was calculated (C). Error bars represent SE (n=3). Asterisks indicate significance levels when comparing to ZH11 in *t*-test (*, P<0.05; **, P<0.01).
