# Supplementary material for: The NLP-HSF regulatory module contributes to nitrogen-mediated thermotolerance in rice
Source: Plant Commun. 2025 Sep 8;6(11):101522. doi: 10.1016/j.xplc.2025.101522 (PMC12785025; doi:10.1016/j.xplc.2025.101522)
Supplement: Document S3. Article plus supplemental information [file mmc3.pdf]

Plant Communications
Correspondence

CellPress
Partner Journal

The NLP-HSF regulatory module contributes to nitrogen-mediated thermotolerance in rice

Dear Editors,

High-temperature stress poses a significant threat to global crop productivity, particularly in staple crops such as rice (*Oryza sativa)*, wheat (*Triticum aestivum)*, and maize (*Zea mays)*. Models predict that global yields of these crops will decline by 3%–8% for every 1°C increase in mean temperature. Over the past two decades, much progress has been made in elucidating the molecular mechanisms underlying plant responses to extreme temperatures, including signal perception, transduction, and transcriptional regulation (Li et al., 2023). As climate change intensifies, developing heat-resilient crops has become a critical challenge for sustainable agriculture. Nitrogen (N), an essential macronutrient, is fundamental to plant growth, metabolism, and stress responses (Liu et al., 2022b). Beyond its nutritional role, N also acts as a signaling molecule that modulates growth and development (Liu et al., 2022b). NIN-like proteins (NLPs), first identified as regulators of nodulation in legumes, are now recognized as master regulators of nitrate (NO_3−N) signaling and metabolism (Liu et al., 2017, 2022a). In rice, NLP3 translocates from the cytoplasm to the nucleus in response to NO_3−N, where it binds NO_3−N-responsive *cis*-elements in the promoters of genes involved in N uptake and assimilation, thereby orchestrating their expression (Zhang et al., 2022b). Field studies further show that heat stress reduces photosynthesis and nitrogen use efficiency (NUE), whereas supplemental N enhances heat tolerance (Wang et al., 2008). However, the molecular basis of this effect remains unclear.

In this study, we show that the N sensor NLP3 plays a critical role in rice thermotolerance, particularly under low N conditions. NLP3 translocates to the nucleus in response to heat stress, where it modulates the expression of heat shock factors (Hsfs). These findings highlight the interplay between N nutrition and thermotolerance in rice, providing novel insights into the integration of nutrient and stress responses in plants.

To examine the role of N in rice thermotolerance, we grew wild-type ZH11 under different concentrations of mixed N supply (0.02 mM, 0.2 mM, 2 mM, 1:1 NO_3^-:NH_4^+) and assessed their sensitivity to heat stress. Before treatment, plants showed similar height (Supplemental Figure 1A). After exposure to heat stress (45°C for 3 days) followed by recovery at 29°C for 7 days, plants grown under low N were more sensitive to heat stress (Supplemental Figure 1B), and the survival rates were significantly reduced when N was limited (Figures 1A–1F; Supplemental Figure 1C). These results indicate that adequate N nutrition is essential for enhancing thermotolerance in rice.

To understand how N affects thermotolerance, we performed RNA sequencing (RNA-seq) to compare heat stress responses under N deficiency (LN, 0.2 mM, mixed N supply) and N sufficiency (NN, 2 mM, mixed N supply). We identified 73 genes induced by heat stress under both conditions, with differential expression between N sufficiency and N deficiency at 45°C but not at 29°C (Supplemental Figures 1D and 1E; Supplemental Data 1). GO and KEGG analyses showed significant enrichment of terms including "response to temperature stimuli," "response to heat," "protein processing in endoplasmic reticulum," and "starch and sucrose metabolism" (Supplemental Figure 2). To validate the RNA-seq results, we selected eight *Hsf* genes from this group and performed quantitative reverse-transcription PCR (RT-qPCR). All eight genes were upregulated by heat stress under both N supply conditions, but induction was much stronger under N sufficiency (Supplemental Figure 3). These results indicate that adequate N is required for robust heat stress responses in rice.

The NLP family of transcription factors acts as transcriptional activators that regulate downstream genes involved in N uptake and assimilation. The rice genome encodes six *NLP* genes, and we examined their expression in response to heat stress under different mixed N supply conditions. None of the *NLP* genes were altered by heat stress (Supplemental Figure 4). Because NLP3 responds to N supply and serves as the major NLP regulating NUE and grain yield in rice (Zhang et al., 2022b), we focused on NLP3 in subsequent analyses. We obtained *NLP3* gene-edited mutants (*nlp3-1* and *nlp3-2*; Supplemental Figure 5) and *NLP3* overexpression lines (*NLP3OE-1* and *NLP3OE-10*) (Zhang et al., 2022b) for phenotypic analysis. Under low N conditions (0.2 mM, mixed N supply), the survival rate of *NLP3* mutants was lower than that of ZH11 plants after heat stress and recovery (Figures 1A–1C), whereas the survival rate of *NLP3* overexpression plants was higher than that of ZH11 (Figures 1G–1I). Under N sufficiency (2 mM, mixed N supply), survival rates did not differ among genotypes (Figures 1D–1F and 1J–1L). After heat stress, N content in both shoots and roots was lower in *NLP3* mutants but higher in *NLP3* overexpression plants compared with ZH11 (Supplemental Figure 6), underscoring the importance of N in thermotolerance. ^{15}N-NO_3^- uptake assays further showed that NO_3−N acquisition was impaired in the *nlp3* mutants in both shoots and roots under heat stress (Supplemental Figure 7). Together, these results demonstrate that NLP3 is required for N-mediated thermotolerance in rice.

NO_3^- and ammonium (NH_4^+) are the two primary N sources assimilated by plants. To determine which form of N contributes to thermotolerance in rice, we grew ZH11, *nlp3-1*/*nlp3-2* mutants, and *NLP3OE-1*/*NLP3OE-10* overexpression lines in media supplemented with either NO_3^- or NH_4^+ as the sole N source, followed by thermotolerance assays. When grown with 1 mM NO_3−N, the *nlp3-1*/*nlp3-2* mutants showed significantly greater heat sensitivity than ZH11, whereas the *NLP3OE-1*/*NLP3OE-10* lines displayed enhanced thermotolerance (Supplemental Figures 8A–8D). In contrast, when 1 mM NH_4^+ was supplied, no

© 2025 The Author(s). Published by Elsevier Inc. on behalf of CAS Center for Excellence in Molecular Plant Sciences, Chinese Academy of Sciences, and Chinese Society for Plant Biology.
This is an open access article under the CC BY license (http://creativecommons.org/licenses/by/4.0/).

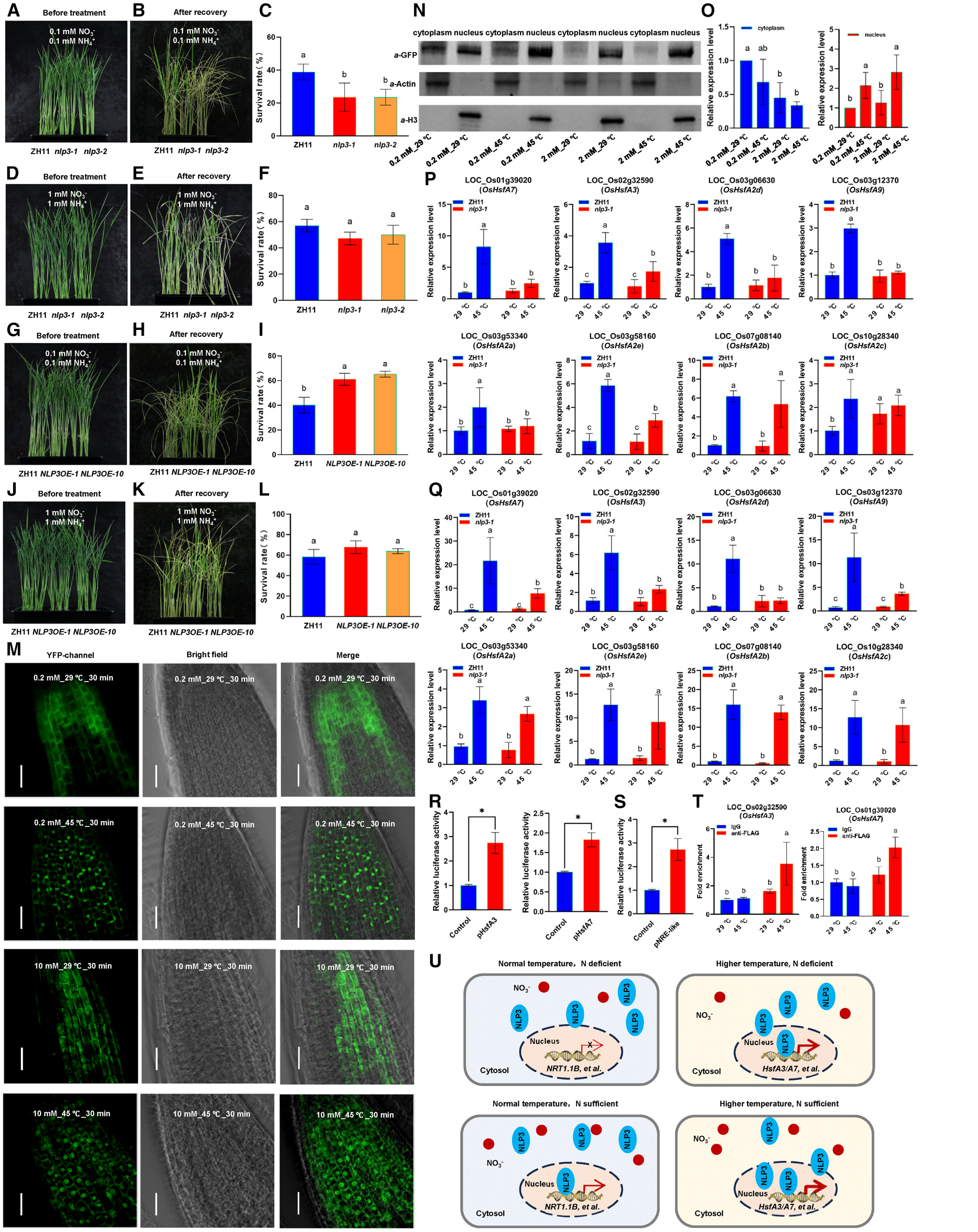

Figure 1. The NLP-HSF module is involved in nitrogen-mediated thermotolerance

(A–L) Phenotypic analysis. Wild-type ZH11, *nlp3* mutant, and *NLP3* overexpression plants grown under different mixed N conditions at 29°C were subjected to heat stress (45°C, 3 d) and then recovered at 29°C for 7 d. Plants were photographed, and survival rates were calculated.

(legend continued on next page)

differences in thermotolerance were observed between ZH11 and either the *nlp3* mutants or the *NLP3OE* overexpression lines (Supplemental Figures 8E–8H). These results indicate that NO_3^-, rather than NH_4^+, plays a critical role in NLP3-mediated thermotolerance in rice.

NLP proteins relocate from the cytoplasm to the nucleus in response to transient N signals (Liu et al., 2017). To examine this process, we developed NLP3-GFP plants and observed the subcellular localization of NLP3-GFP in rice roots under different temperature conditions. Plants grown under N-deficient conditions (0.2 mM, mixed N supply) at 29°C for 7 days were subjected to heat stress (45°C) and N treatment (10 mM) for 30 min. Nuclear GFP signals in roots were more evident under heat stress conditions than under normal temperature (Figure 1M). We also grew NLP3-GFP plants under both N-deficient (0.2 mM, mixed N supply) and N-sufficient (2 mM, mixed N supply) conditions for 7 days and then exposed them to heat stress (45°C) for 2 h. Cytoplasmic and nuclear fractions were isolated from whole seedlings and analyzed by Western blotting. NLP3-GFP accumulated more strongly in the nucleus in response to both N supply and heat stress, with the highest levels under combined N sufficiency and heat stress (Figures 1N and 1O). These results indicate that NLP3 relocates from the cytoplasm to the nucleus under heat stress.

To understand how nucleus-localized NLP3 regulates thermotolerance, we examined the expression of the eight *Hsf* genes mentioned above in ZH11 and *nlp3-1* mutant plants under different N and temperature conditions. Loss of *NLP3* function suppressed the expression of *HsfA7*/A3/A2d/*A9* under both 0.2 mM N and 2 mM N conditions (Figures 1P and 1Q) but did not affect the expression of *NIA1*/*NIA2*/*NRT2.4* (Supplemental Figure 9). Expression of *NRT1.1B* was upregulated by heat stress in ZH11 plants; however, this induction was impaired in the *nlp3-1* mutant (Supplemental Figure 9). To test whether NLP3 directly regulates *HsfA3/A7/A2d*, we performed effector-reporter assays using their 1.5 kb promoter sequences fused to firefly luciferase (Supplemental Figure 10A). NLP3 activated promoter-driven luciferase activity for *HsfA3/A7/A2d* (Figure 1R; Supplemental Figure 10B), indicating possible direct regulation. Because NLP3 was previously shown to bind to the NO_3^--responsive-like (NRE-like) *cis*-elements, we further tested this using an effector-reporter assay with the TTGACC motif from the *HsfA7* promoter. NLP3 indeed activated this NRE-like *cis*-element (Figure 1S). Chromatin immunoprecipitation (ChIP)-qPCR confirmed that NLP3 directly binds the promoter regions of *HsfA3/A7* but not *HsfA2d* under heat stress (Figure 1T; Supplemental Figure 10C). These results demonstrate that NLP3 activates the expression of *HsfA3/A7* under heat stress.

To investigate the functional role of HsfA3 in thermotolerance, we generated two independent mutant lines, *hsfa3-1*/*hsfa3-2* (Supplemental Figures 11A and 11B), and performed phenotypic analysis. These mutants were more sensitive to heat stress than ZH11 plants (Supplemental Figure 12), suggesting that the downstream target gene *HsfA3* is important for thermotolerance in rice.

N is a critical macronutrient for plants, and emerging evidence suggests its involvement in diverse abiotic stress responses, including ionic and drought stress (Liu et al., 2022b). Plants mainly absorb N in the form of NO_3^- and NH_4^+. In rice, the high-affinity NO_3^- transporter NRT2.3 exists as two splice variants (NRT2.3a and NRT2.3b) and plays a pivotal role in NUE and yield (Zhang et al., 2022a). Notably, *NRT2.3a* expression is negatively regulated by the temperature-dependent small RNA *sNRT2.3-1*, whereas rice accessions carrying the *high-temperature-resistant and nitrogen-efficient-2* (*HTNE-2*) allele show enhanced *NRT2.3b* translation and improved yield under high nighttime temperatures (Zhang et al., 2022a). These findings establish a direct link between N nutrition and temperature in rice.

In this study, we examined the role of N nutrition in heat stress response and demonstrated that NLP3, a NO_3^- sensor and regulator, integrates N nutrition (especially NO_3^-) with thermotolerance in rice by directly regulating *Hsf*s and other heat-responsive genes (Figure 1U). Our findings establish that optimal N nutrition is essential for rice thermotolerance. However, excessive N fertilization can compromise grain quality and contribute to environmental degradation (Liu et al., 2022b). Over the past two decades, major progress in understanding the molecular mechanisms governing NUE in crops, particularly rice, has opened opportunities to enhance thermotolerance through genetic improvement of NUE rather than relying solely on increased fertilizer use. This strategy is particularly promising in high-NUE rice varieties that maintain robust thermotolerance even under low N input conditions.

(**M**) Subcellular localization. Seven-day-old *NLP3-GFP* plants pre-cultured under low N (0.2 mM) were treated with high N (10 mM KNO_3) for 30 min under either normal (29°C) or heat stress (45°C) conditions. Root samples were observed by confocal microscopy. Scale bar, 50 μm.

(N and O) Cytoplasm-nucleus fractionation. *NLP3-GFP* plants grown under low N (0.2 mM) or normal N (2 mM) were subjected to 45°C for 2 h. Seedlings were fractionated into cytoplasmic and nuclear components, and NLP3-GFP was detected by Western blotting with anti-GFP. Anti-Actin and anti-H3 served as cytoplasmic and nuclear markers, respectively. Band intensities were quantified from three independent blots.

(P and Q) Gene expression analysis. Plants grown under 0.2 mM **(P)** or 2 mM **(Q)** mixed N conditions at 29°C were subjected to 45°C treatment for 2 h and analyzed by RT-qPCR.

(R and S) Effector-reporter assays. Promoter sequences of *HsfA3/A7* or the NRE-like motif (TTGACC) were fused to firefly luciferase as reporters, and constitutively expressed NLP3 served as the effector. Renilla luciferase driven by the 35S promoter was used as an internal control. Relative luciferase activity was calculated by normalizing firefly to Renilla activity and then to the empty vector control.

(T) ChIP-qPCR. Fourteen-day-old *NLP3-FLAG* overexpression plants grown at 29°C under normal N were subjected to 45°C for 2 h and analyzed by ChIP-qPCR. Error bars represent SE (n = 3). Asterisks indicate significance compared with the control in t-test (*p < 0.05). Different letters denote significant differences based on Tukey's HSD test (p < 0.05).

(U) Working model for NLP3. Under normal temperatures, NLP3 translocates from the cytoplasm to the nucleus in response to NO_3–N supply, where it regulates N-responsive genes such as *NRT1.1B*. Under heat stress, NLP3 similarly relocates to the nucleus but instead activates a distinct set of heat-responsive genes, including *HsfA3/A7*. N supply promotes nuclear accumulation of NLP3 and enhances heat-responsive gene expression, highlighting the critical role of N nutrition in plant thermotolerance.

Rice encodes six *NLP* genes, among which *NLP1*, *NLP3*, and *NLP4* synergistically regulate N utilization (Wu et al., 2021; Zhang et al., 2022b). Our phenotypic analyses show that NLP3, the rice ortholog of *Arabidopsis* NLP7, plays an essential role in thermotolerance, particularly under N-limited conditions. Both *nlp3* mutants and *NLP3*-overexpressing lines exhibited strong thermotolerance phenotypes at 0.2 mM (mixed N) and 1 mM N (pure NO_3^-) but not at 2 mM (mixed N), indicating that N status is important for the manifestation of thermotolerance in rice. Notably, *nlp3* mutants displayed impaired heat-responsive gene expression under N sufficiency, and NLP3 protein translocated to the nucleus under heat stress regardless of N availability. These results suggest that while NLP3 contributes significantly to thermotolerance, functional redundancy among NLP family members may compensate for *NLP3* loss under some conditions. Further studies are needed to clarify the cooperative roles of other NLPs in rice thermotolerance.

Heat shock factors (Hsfs) are master regulators of HSPs and other thermotolerance-related genes. The rice genome contains 25 *Hsf* genes, and overexpression of *HSFA2e* confers thermotolerance even in *Arabidopsis* (Guo et al., 2016). We found that NLP3 directly regulates the expression of *HsfA3* and *HsfA7*; however, a previous study reported that *HsfA7* overexpression does not enhance thermotolerance in rice (Liu et al., 2013). Our results show that *nlp3* mutants have significantly reduced induction of key *Hsf*s, including *HsfA2d*, *HsfA3*, *HsfA7*, and *HsfA9*, under heat stress. This suggests that other *Hsf* genes besides *HsfA7* function redundantly downstream of NLP3 to confer thermotolerance. Indeed, we confirmed that HsfA3 is critical for thermotolerance in rice (Supplemental Figure 12). These findings establish NLP3 as a critical regulator of *Hsf* gene expression during the heat stress response. In addition, we observed that N nutrition also modulates the expression of heat-responsive genes beyond the *Hsf* family (Supplemental Data 1), suggesting that NLP3 regulates thermotolerance through both *Hsf*-dependent and *Hsf*-independent pathways. Future transcriptomic comparisons between ZH11 and *nlp3* mutants under varying N and temperature conditions will further clarify the role of NLP3 in thermotolerance.

In summary, our study identifies a previously uncharacterized pathway in which NLP3 mediates thermotolerance by regulating heat-responsive genes, thereby expanding the functional interplay between N nutrition and heat stress adaptation in rice.

FUNDING
This project was financially supported by the State Key Project of Research and Development Plan, China (grant no. 2021YFF1000404).

ACKNOWLEDGMENTS
The authors declare no conflict of interest.

AUTHOR CONTRIBUTIONS
D.-J.Z. and J.-X.L. designed the experiments; D.-J.Z., Z.-S.Z., T.Q., and J.G. performed the experiments; J.-X.L., D.-J.Z., and C.-B.X. analyzed the data; J.-X.L. and D.-J.Z. wrote the paper.

SUPPLEMENTAL INFORMATION
Supplemental information is available at *Plant Communications Online*.

Received: June 25, 2025
Revised: August 24, 2025
Accepted: September 5, 2025
Published: September 8, 2025

Dong-Jie Zhu[1], *Zi-Sheng Zhang*[2], *Tao Qing*[1], *Juan Gao*[1], *Cheng-Bin Xiang*[2] *and Jian-Xiang Liu*[1,*]

[1]State Key Laboratory of Plant Environmental Resilience, College of Life Sciences, Zhejiang University, Hangzhou 310027, China
[2]School of Life Sciences, University of Science and Technology of China, Hefei 230027, China
***Correspondence: Jian-Xiang Liu (jianxiangliu@zju.edu.cn)**
https://doi.org/10.1016/j.xplc.2025.101522

REFERENCES
Guo, M., Liu, J.H., Ma, X., Luo, D.X., Gong, Z.H., and Lu, M.H. (2016). The plant heat stress transcription factors (HSFs): Structure, regulation, and function in response to abiotic stresses. Front. Plant Sci. **7**:114.

Li, J.Y., Yang, C., Xu, J., Lu, H.P., and Liu, J.X. (2023). The hot science in rice research: How rice plants cope with heat stress. Plant Cell Environ. **46**:1087–1103.

Liu, A.L., Zou, J., Liu, C.F., Zhou, X.Y., Zhang, X.W., Luo, G.Y., and Chen, X.B. (2013). Over-expression of *OsHsfA7* enhanced salt and drought tolerance in transgenic rice. BMB Rep. **46**:31–36.

Liu, K.H., Liu, M., Lin, Z., Wang, Z.F., Chen, B., Liu, C., Guo, A., Konishi, M., Yanagisawa, S., Wagner, G., et al. (2022a). NIN-like protein 7 transcription factor is a plant nitrate sensor. Science **377**:1419–1425.

Liu, K.H., Niu, Y., Konishi, M., Wu, Y., Du, H., Sun Chung, H., Li, L., Boudsocq, M., McCormack, M., Maekawa, S., et al. (2017). Discovery of nitrate-CPK-NLP signalling in central nutrient-growth networks. Nature **545**:311–316.

Liu, Q., Wu, K., Song, W., Zhong, N., Wu, Y., and Fu, X. (2022b). Improving crop nitrogen use efficiency toward sustainable green revolution. Annu. Rev. Plant Biol. **73**:523–551.

Wang, D., Heckathorn, S.A., Mainali, K., and Hamilton, E.W. (2008). Effects of N on plant response to heat-wave: A field study with prairie vegetation. J. Integr. Plant Biol. **50**:1416–1425.

Wu, J., Zhang, Z.S., Xia, J.Q., Alfatih, A., Song, Y., Huang, Y.J., Wan, G. Y., Sun, L.Q., Tang, H., Liu, Y., et al. (2021). Rice NIN-LIKE PROTEIN 4 plays a pivotal role in nitrogen use efficiency. Plant Biotech. J. **19**:448–461.

Zhang, Y., Tateishi-Karimata, H., Endoh, T., Jin, Q., Li, K., Fan, X., Ma, Y., Gao, L., Lu, H., Wang, Z., et al. (2022a). High-temperature adaptation of an *OsNRT2.3* allele is thermoregulated by small RNAs. Sci. Adv. **8**:eadc9785.

Zhang, Z.S., Xia, J.Q., Alfatih, A., Song, Y., Huang, Y.J., Sun, L.Q., Wan, G.Y., Wang, S.M., Wang, Y.P., Hu, B.H., et al. (2022b). Rice *NIN-LIKE PROTEIN 3* modulates nitrogen use efficiency and grain yield under nitrate-sufficient conditions. Plant Cell Environ. **45**:1520–1536.

Plant Communications, Volume *6*

Supplemental information

The NLP-HSF regulatory module contributes to nitrogen-mediated thermotolerance in rice

Dong-Jie Zhu, Zi-Sheng Zhang, Tao Qing, Juan Gao, Cheng-Bin Xiang, and Jian-Xiang Liu

Supplemental information

The NLP-HSF regulatory module contributes to nitrogen-mediated thermotolerance in rice

Dong-Jie Zhu[1], Zi-Sheng Zhang[2], Tao Qing[1], Juan Gao[1], Cheng-Bin Xiang[2] and Jian-Xiang Liu[1,*]

[1]State Key Laboratory of Plant Physiology and Biochemistry, College of Life Sciences, Zhejiang University, Hangzhou 310027, China.

[2] School of Life Sciences, University of Science and Technology of China, Hefei 230027, China.

[*]Correspondence：jianxiangliu@zju.edu.cn.

Supplemental information

Materials and methods

Plant materials and genetic constructs

The wild-type plants used in this study were of the ZH11 background. To generate gene-edited mutants, gene-specific guide sequences (sgRNAs) were designed, and two mutant alleles (*nlp3-1*, *nlp3-2*) were obtained using CRISPR-Cas9 technology (Hangzhou Biogle Co., Ltd, Hangzhou, China). Two *NLP3* overexpression lines (*NLP3OE-1*, *NLP3OE-10*) were generated by cloning the full-length coding sequence (CDS) of *NLP3* into the pCB2006 vector with the constitutive *Actin1* promoter, which was then introduced into ZH11 via *Agrobacterium*-mediated transformation. All primers used are listed in **Table S1**.

Phenotypic analysis under different nitrogen conditions

Seeds were soaked in water at room temperature for 48 hours, followed by germination at 37°C. The germinated seeds were then transferred to black boxes containing modified Kimura B solution. The basic modified Kimura B solution (2 mM) consisted of the following macronutrients: $(NH_4)_2SO_4$ (0.5 mM), KNO_3 (1 mM), $MgSO_4 \cdot 7H_2O$ (0.54 mM), $CaCl_2$ (0.36 mM), K_2SO_4 (0.09 mM), KH_2PO_4 (0.18 mM), and $Na_2SiO_3 \cdot 9H_2O$ (0.7 mM); and micronutrients: $MnCl_2 \cdot 4H_2O$ (9.14 μM), H_3BO_3 (46.2 μM), $Na_2MoO_4 \cdot 2H_2O$ (0.56 μM), $ZnSO_4 \cdot 7H_2O$ (0.76 μM), $CuSO_4 \cdot 5H_2O$ (0.32 μM), and Fe(II)-EDTA (40 μM). For the 0.02 mM and 0.2 mM mixed N treatments, all nitrogen sources were replaced with KNO_3 and NH_4Cl provided as a 1:1 molar ratio of NO_3^- : NH_4^+. For ammonium-only treatment, all nitrogen sources were removed from the modified Kimura B solution and substituted with 1 mM NH_4Cl as the sole nitrogen source. For nitrate-only treatment, all nitrogen sources were eliminated from the modified Kimura B solution and replaced with 1 mM KNO_3 as the exclusive nitrogen source. The nutrient solution was refreshed every two days. The pH was adjusted to 5.5 using HCl.

Growth conditions and heat stress treatment

Plants were cultivated under 20,000 lux light conditions (12-h light/12-h dark cycle) in growth chambers (CONVIRON PGR15). Heat stress was induced by transferring 7-day-old plants from 29°C to a growth chamber set at 45°C for 2–3 days. Subsequently, the plants were returned to 29°C for a 7-day recovery period [1]. Representative plants were photographed, and survival rates were calculated for each experiment.

RNA-seq analysis

For RNA-seq, 7-day-old ZH11 seedlings grown at 29°C under different mixed nitrogen levels (0.2 mM or 2 mM) were subjected to a 2-hour heat treatment (45°C) before sampling. Sequencing was performed on an Illumina HiSeq 4000 platform (LC-Bio Technologies) following standard Illumina protocols [2]. Clean reads were aligned to the reference genome using HISAT2 (v2.2.1; https://daehwankimlab.github.io/hisat2/), and transcript assembly was conducted with StringTie (v2.1.6; http://ccb.jhu.edu/software/stringtie/) using default parameters. Gene expression levels were normalized as fragments per kilobase of transcript per million mapped reads (FPKM). Differentially expressed genes (DEGs) were identified by: DESeq2 (v1.40.2) for pairwise group comparisons. edgeR (v3.42.4) for individual sample comparisons. Genes with $q < 0.05$ and |fold change (FC)| ⩾ 2 were classified as upregulated, while those with $q < 0.05$ and FC ⩽ 0.5 were considered downregulated. GO (Gene Ontology) and KEGG pathway analyses were performed using clusterProfiler (v4.2.2). The top 10 enriched pathways (ranked by P-value) were visualized as bubble plots using ggplot2 (v3.5.1) on the OmicStudio cloud platform (LC-Bio Technology). Statistical significance was assessed using a two-sided test (no multiple-testing correction applied). The RNA-seq data is deposited in the Genome Sequence Archive (GSA) under the accession number (CRA026108).

RT-qPCR analysis

Total RNA was extracted using the RNA Prep Pure Plant Kit (Tiangen, Shanghai,

China) and reverse-transcribed into cDNA with the Evo M-MLV Reverse Transcription Reagent Premix (Accurate Biology, Hangzhou, China). Quantitative real-time PCR (RT-qPCR) was performed using the SuperReal Premix Color kit (Tiangen, Beijing, China) on a CFX96 Real-Time System (Bio-Rad, Hercules, CA, USA). Relative gene expression levels were calculated using the ΔΔCt method. *PP2A* was used as the internal reference gene for normalization. The expression level of the first treatment sample was set to 1, and the relative expression of other samples was calculated accordingly.

^{15}N-nitrate uptake and nitrogen content measurement

^{15}N-uptake assay was used with ^{15}N nitrate-KNO_3 (99 atom % ^{15}N, Macklin, C11510585). For ^{15}N-nitrate uptake assay, 7-day-old wild type (ZH11), mutant (*nlp3*), and OE plants (*NLP3-GFP*) were cultured in the Kimura B solution for 7 days. The plants were pre-treated with the fresh modified Kimura B containing 0.1 or 1 mM ^{15}N-KNO_3 solution for 2 hr, and then transferred from 29°C to 45°C for 2 d. The roots were washed for 1 min in 0.1 mM $CaSO_4$.Then the samples were analyzed for nitrogen content measurement. Samples were collected, dried in an oven, and ground into fine powder using a grinding mill (120 s). Approximately 1 mg of each powdered sample was weighed and analyzed for nitrogen content using an isotope ratio mass spectrometer (IRMS; Thermo Finnigan MAT DELTA plus XP).

Subcellular localization assays

Seven-day-old seedlings of *NLP3-GFP* overexpression plants, pre-cultured in modified Kimura B solution containing 0.2 mM nitrogen, were subjected to high nitrogen (10 mM) KNO_3 treatment for 30 min under either normal (29°C) or heat stress (45°C) conditions. After treatment, root samples were immediately collected for confocal imaging using a two-photon laser scanning microscope (LSM710nlo, Carl Zeiss, Oberkochen, Germany) to visualize NLP3-GFP subcellular localization.

Subcellular protein fractionation assays

Nuclear-cytoplasmic fractionation was carried out following previous published

paper [3]. Seven-day-old seedlings of *NLP3-GFP* overexpression plants grown under low N (0.2 mM) and normal N (2 mM) conditions were subjected to 45°C heat stress for 2 hours and then ground into fine powder in liquid nitrogen using a mortar and pestle. The powder was homogenized in Buffer A containing: 5% Triton X-100, 0.25 M sucrose, 10 mM Tris-HCl (pH 7.8), 10 mM $MgCl_2$, 10 mM KCl, 5 mM β-mercaptoethanol, 1 mM PMSF, 1× Roche protease inhibitor cocktail. The homogenate was filtered through Miracloth (Calbiochem) and incubated on ice for 15 min, and centrifuged at 2,000 × g, 4°C, 5 min. The supernatant was collected as the total protein fraction. For cytosolic protein extraction, the supernatant was further centrifuged at 12,000 × g, 4°C, 10 min. The resulting supernatant represented the cytosolic fraction. The pellet from was resuspended in Buffer A, gently pipetted to mix, and centrifuged at 2,000 × g, 4°C, 10 min. This washing step was repeated twice, discarding the supernatant each time. For nuclear protein extraction, the washed pellet was resuspended in Buffer B containing: 25% glycerol, 0.5 mM DTT, 20 mM Tris-HCl (pH 7.5), 2 mM $MgCl_2$, 400 mM NaCl, 0.2 mM EDTA, 1 mM PMSF, 1× Roche protease inhibitor cocktail. The mixture was incubated on ice for 30 min with gentle mixing and centrifuged at 12,000 × g, 4°C, 15 min. The supernatant was collected as the nuclear protein fraction.

Effector-reporter assay

Effector-reporter assays were performed following previous protocol [4]. Briefly, full-length of *NLP3* (CDS) was cloned into pGreenII 62-SK vector to generate the effector, and 1.5 kb promoter sequence of HsfA7 or 4× tandem repeats of NRE-like motif (TTGACC) was inserted into pGreen0800-II vector to make the firefly luciferase reporter, in which Renilla luciferase driven by 35S promoter was used as an internal control. Different combination of vectors was infiltrated into *N. benthamiana* leaves via *Agrobacterium tumefaciens* (GV3101 strain). After 48-72 hours, luciferase was measured with Dual-Luciferase® Reporter Assay System (Promega, Cat.No. E1910).

ChIP–qPCR

For ChIP-qPCR analysis, 7-day-old *NLP3-FLAG* overexpression plants grown at 29ºC under N normal conditions were subjected to heat stress (45ºC) for 2 hr, and then were harvested for fixation with 1% [w/v] formaldehyde for 15 min, which was stopped by adding 0.125 M glycine for 10 min. Nuclei were then extracted and resuspended in a nuclei lysis buffer. After ultrasonication in 0.8% [w/v] SDS buffer, the mix was immunoprecipitated with *anti*-FLAG (Abmart) or the IgG control. The enrichment of DNA fragments was then quantified by qPCR with routine procedures.

[1] Yang C, Luo A, Lu H-P, et al. Diurnal regulation of alternative splicing associated with thermotolerance in rice by two glycine-rich rna-binding proteins. Science Bulletin, 2024, 69: 59-71

[2] Lu HP, Liu XH, Wang MJ, et al. The NAT1-bHLH110-CER1/CER1L module regulates heat stress tolerance in rice. Nature Genetics, 2025, 57:

[3] Kinkema M, Fan WH, Dong XN. Nuclear localization of NPR1 is required for activation of *PR* gene expression. Plant Cell, 2000, 12: 2339-2350

[4] Liu XH, Lyu YS, Yang W, et al. A membrane-associated nac transcription factor OsNTL3 is involved in thermotolerance in rice. Plant Biotechnology Journal, 2020, 18: 1317-1329

Table S1. Primers used in this study

Name	Purpose	Forward Primer (5'-3')	Reverse Primer (5'-3')
NLP3- target 1	sgRNA	GTTATTCCGGTCCACTACCC	CTCGTCGAACAGCCACAG
NLP3- target 2	sgRNA	CTGCTGTTCTCGTCGGTGT	TGCCCCTCTGCTTCAACTTT
pCB2006- NLP3	Overexpression	ATGGAGGTTGACCCATCGTC	TCAACCTGAGCTTCCACAGGAAC
pCB2006- NLP3:GFP	Overexpression	ATGGAGGTTGACCCATCGTC	ACCTGAGCTTCCACAGGAAC
qLOC_Os01g39020	RT-qPCR	GCTTCTTCAAGCACGCCAAC	AGTGACCTTTCGGAACCCGTAG
qLOC_Os01g62290	RT-qPCR	AACACCGTCTTCGATGCCAAGC	GCACCACAATCATAGGCTTATCGC
qLOC_Os02g02410	RT-qPCR	GTTTGAGGTGTTGGCCACCAATG	TCTCTGGTCAAAGTCCTCACCTC
qLOC_Os02g32590	RT-qPCR	GGACCTCAATCTTGCCATGCTG	TGTCGTCTGGTGCTTCGATTCC
qLOC_Os02g52150	RT-qPCR	GTGGTCAAATTCAGGCGGACTC	TAATTGTCTCGCGCACGGTTC
qLOC_Os03g06630	RT-qPCR	GCCAGCTCAACACCTACTTCTTGG	GGCCTCTCAGGAAACCATCATTCG
qLOC_Os03g12370	RT-qPCR	TTACCGAAGCTTGATTGGTGGTC	AGGTTGGTCACCGAAGAACTAATG
qLOC_Os03g53340	RT-qPCR	TCGCCATTCTCACCGACATGAATC	AAGTCGAGCTCCTCCTCTTTCACG
qLOC_Os03g58160	RT-qPCR	TGTGGGATCCTCACCTCTTTGG	TCCACCTTCCTGAAGCCATAGG
qLOC_Os07g08140	RT-qPCR	AGGCAGCTCAACACCTATGGTTTC	TTGCTGGTTTGAAGCAGTATGGG
qLOC_Os10g28340	RT-qPCR	CTCAACACCTACGGCTTCAGAAAG	TGTTGTGACGGAGGTGCATTGG
qPP2A	RT-qPCR	TTATGGGGGATTATGTGGATCG	GTGCTGTCAGTGGAAAATAGTC
qLOC_Os01g13540	RT-qPCR	GCTGGTGAGGTTGATAAAGTCTGC	GCGGCCTTCATTGCATATCTGG
qLOC_Os01g14420	RT-qPCR	ACGCTGAAAGAGAGAAAGCCAAG	CTTGGAAAGCGCCTGGTGATAC
qLOC_Os03g03900	RT-qPCR	CGCAGAAGGTCAACTACAGTGC	GAGCTCCTGAGATCAACTTCCTTG
qLOC_Os04g41850	RT-qPCR	GAGTTGCTTGCCAACTGGACAC	TGGAACTAGGCTGTACCTGCTC
qLOC_Os09g37710	RT-qPCR	AACTTCACCTCCGAGCTCAATACC	GCTTGTCAGGTTAACTGCCTGGAG
qLOC_Os11g16290	RT-qPCR	TCAAGTGGTGGGTCTTGCTGTG	TGCTTAACTGAACAGACTGCAGAG
qLOC_Os08g36480	RT-qPCR	CCAATTCTTTCATCGTGTTCT	CATGCAGCATTTCGTTTCT
qLOC_Os08g36500	RT-qPCR	ACTGGTGCTGGTGCTTCTGG	CGGCTGGGTGTTGAGGGACT
qLOC_Os10g40600	RT-qPCR	GGCAGGCTCGACTACTTCTA	AGGCGCTTCTCCTTGTAGAC
qLOC_Os01g36720	RT-qPCR	TTCGTCGCGCTCCGGTTCG	CGCACGGGAGTAGGTAGGTG
35S-NLP3	Effector	TCCCCGCGGATGGAGGTTGACCCATCGTC	CGGGATCCTCAACCTGAGCTTCCACAGG
pNRE-like	Reporter	TCGACATTGACCTTGACCTTGACCTTGACC	AGCTGGTCAAGGTCAAGGTCAAGGTCAATG
pHSFA7	Reporter	GCGTCGACAGTGAGGCTCTTGTAACC	CCAAGCTTCGGTGGCAATGCATGGCT
pHSFA3	Reporter	GCGTCGACACCGATCGAGCCAAGCCA	CCAAGCTTGATATGGATTGCGGGGGG
pHSFA2d	Reporter	GCGTCGACGAGTGTTTATATACAATAA	CCAAGCTTAATATAAACAACCGTGTA
q-pHSFA7	ChIP-qPCR	CCCTCCGTCCACAGTTTTAC	CCGTGTCCTACGTTTGATCG
q-pHSFA3	ChIP-qPCR	TTCTGAACCTCTATTTTGGC	TAACATCAATATGAATGTGG
q-pHSFA2d	ChIP-qPCR	ATATCGTGTCTGGTAGAATC	CGGTGATTGGCACACACGA

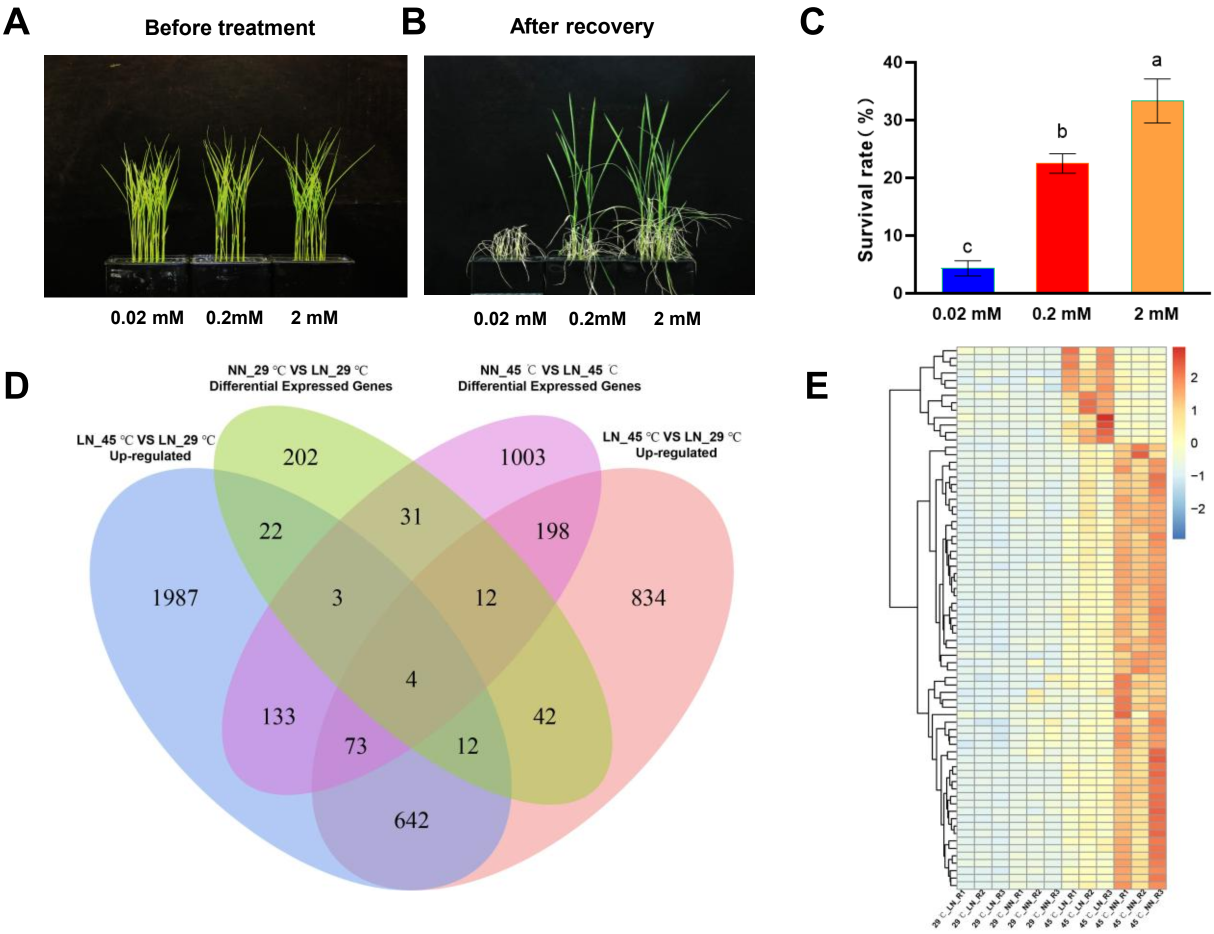

Figure S1. Enhancing heat stress tolerance by nitrogen nutrition. A-C, Phenotypic analysis. Wild-type ZH11 plants cultivated in different nitrogen concentration (1:1 NO_3^-:NH_4^+) at 29°C were subjected to heat stress (45°C) treatment for 3 d and then recovered at 29°C for 7 d, and plants were photographed (A-B) and survival rate (C) was calculated. **D-E**, Transcriptional analysis. ZH11 plants grown under 0.2 mM (LN) or 2 mM (NN) nitrogen conditions at 29°C were subjected to heat stress (45°C) for 2 hr and harvested for RNA-seq analysis (D). The expression of 73 genes involved in nitrogen-mediated heat responses is shown with heat map (E). Error bars represent SE (n = 3). Different letters indicate significant differences as determined by a Tukey's HSD test ($P < 0.05$).

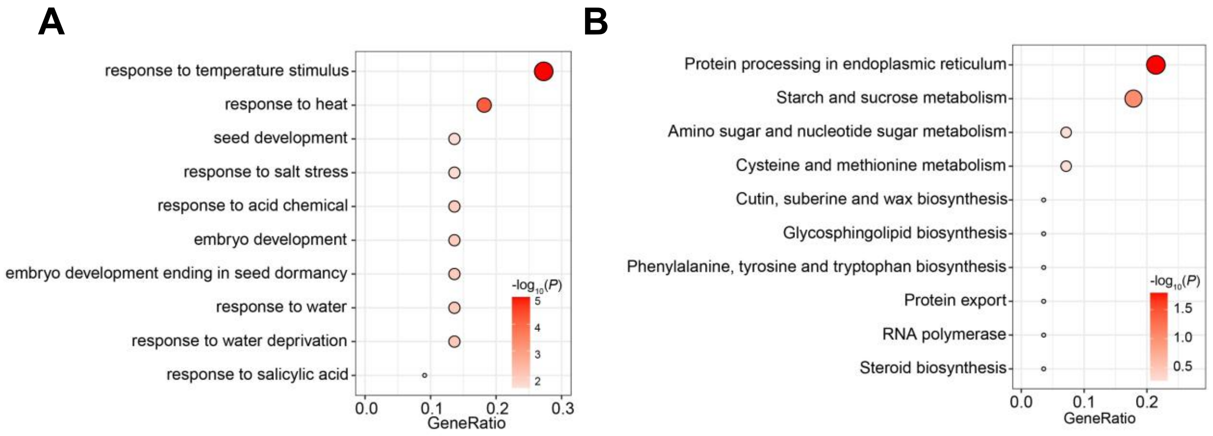

Figure S2. GO and KEGG analysis of N-dependent heat responsive genes. Wild-type ZH11 plants grown under 0.2 mM and 2 mM nitrogen conditions at 29°C were subjected to heat stress (45°C) for 2 hr and harvested for RNA-seq analysis. Totally 73 heat responsive genes related to N supply were used for GO (A) KEGG (B) analysis.

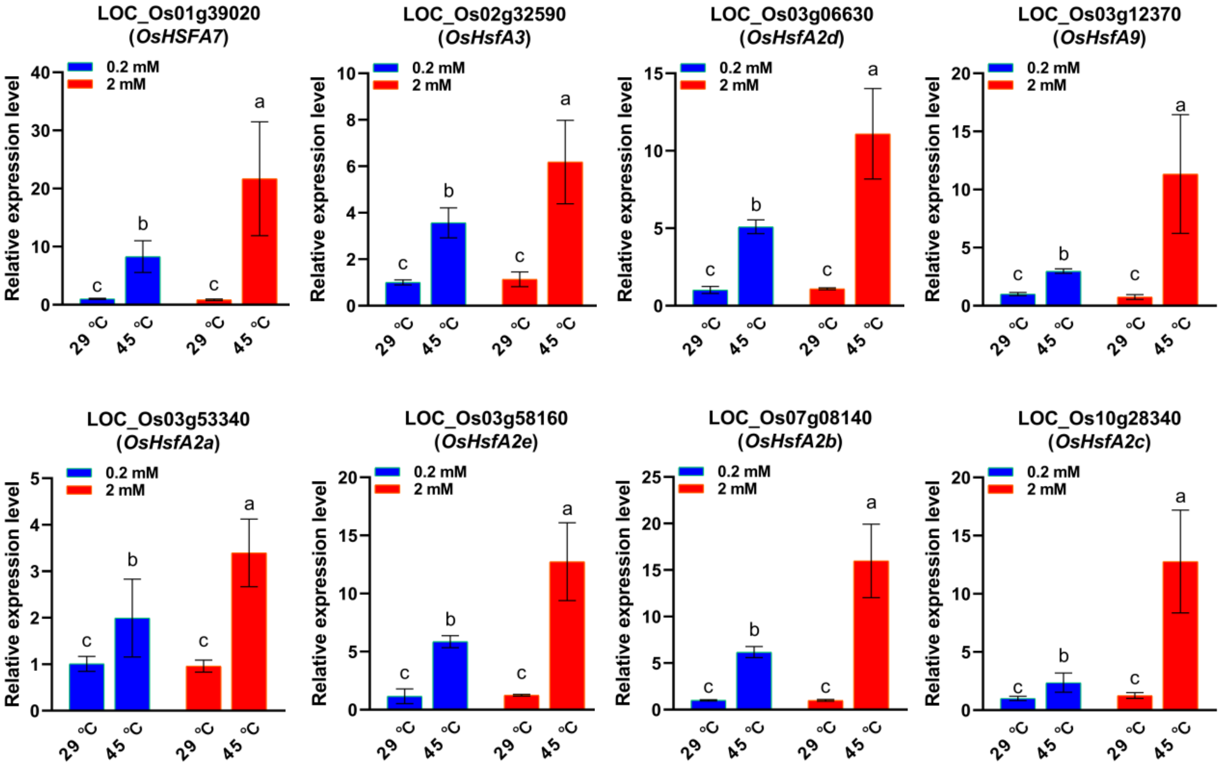

Figure S3. Validation of RNA-Seq analysis. Wild-type ZH11 plants grown under different nitrogen conditions at 29°C were subjected to heat stress (45°C) for 2 hr and harvested for RT-qPCR. Error bars represent SE (n = 3). Different letters indicate significant differences as determined by a Tukey's HSD test ($P < 0.05$).

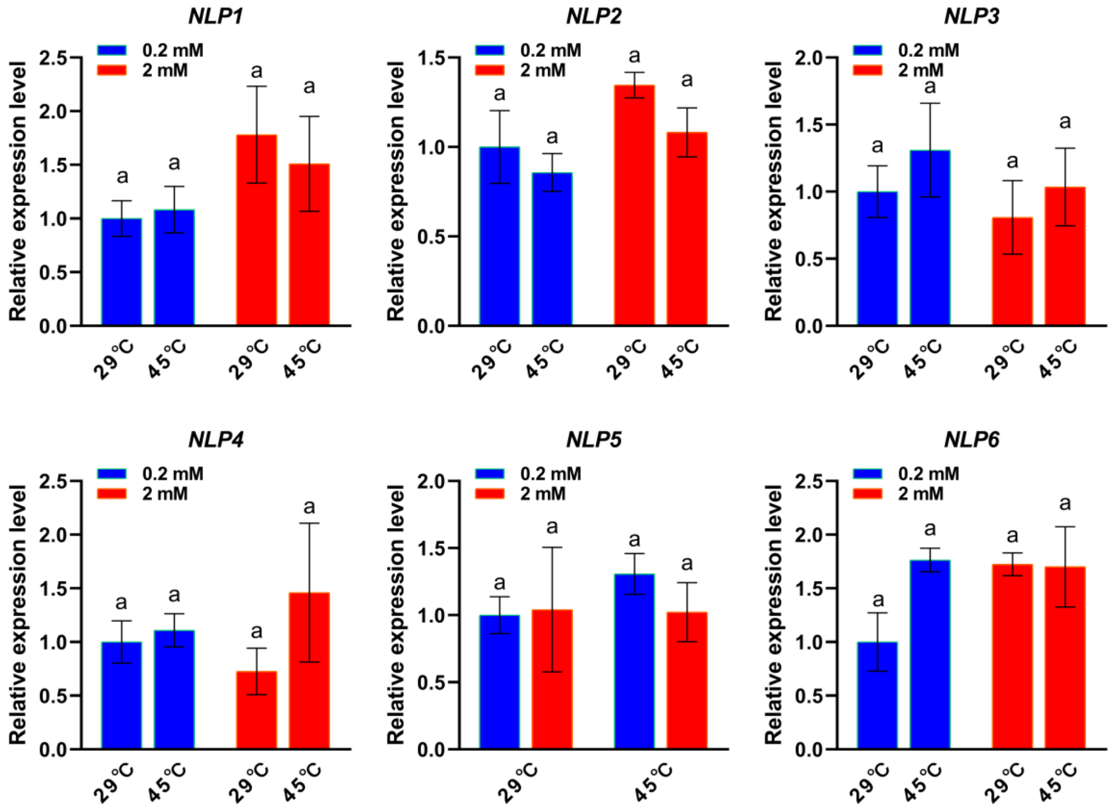

Figure S4. Responses of *NLP1-6* genes to heat stress. ZH11 plants grown under 0.2 mM and 2 mM nitrogen conditions at 29°C were subjected to heat stress (45°C) for 2 hr and harvested for RNA-seq analysis. Error bars represent SE (n = 3). Different letters indicate significant differences as determined by a Tukey's HSD test ($P < 0.05$).

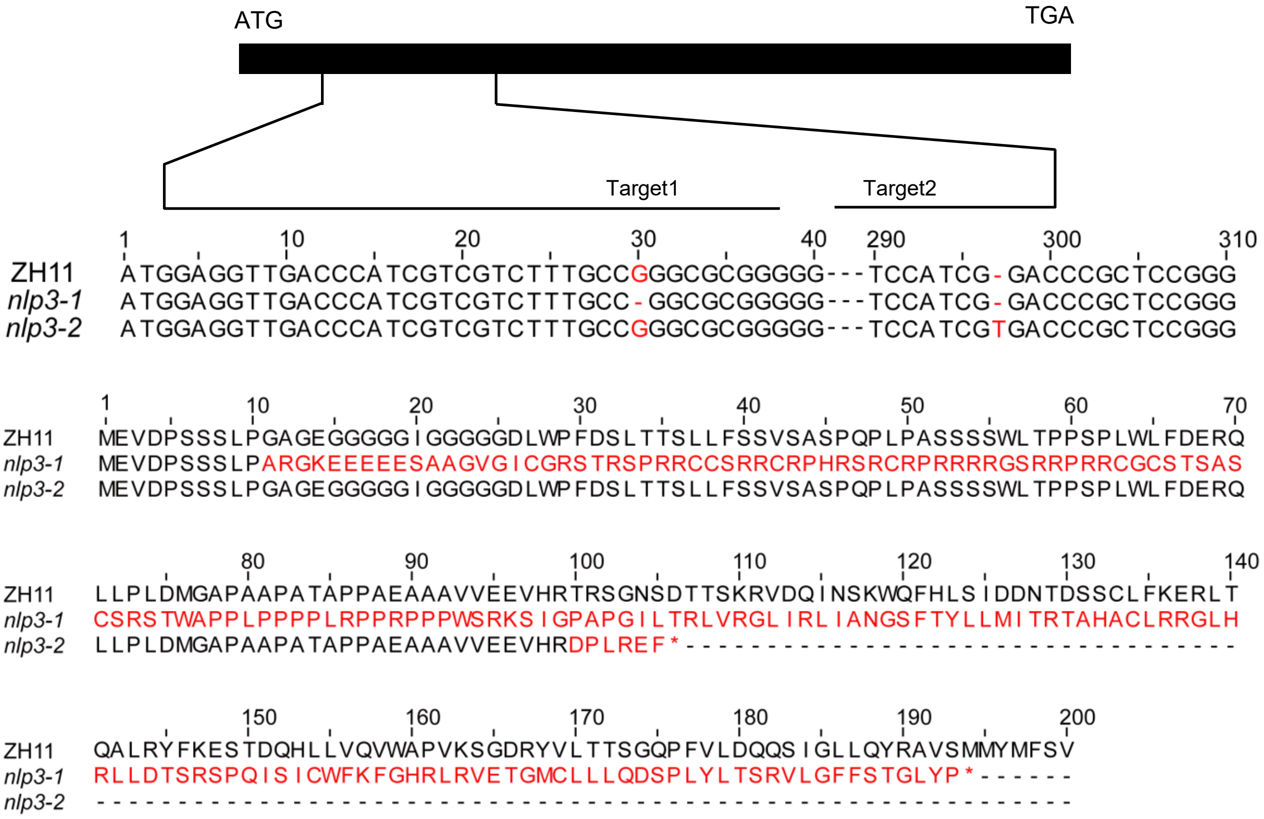

Figure S5. Characterization of *nlp3* mutants. The mutated nucleic acids and protein sequences of NLP3 in wild-type ZH11 and gene-edited mutants (*nlp3-1/-2*).

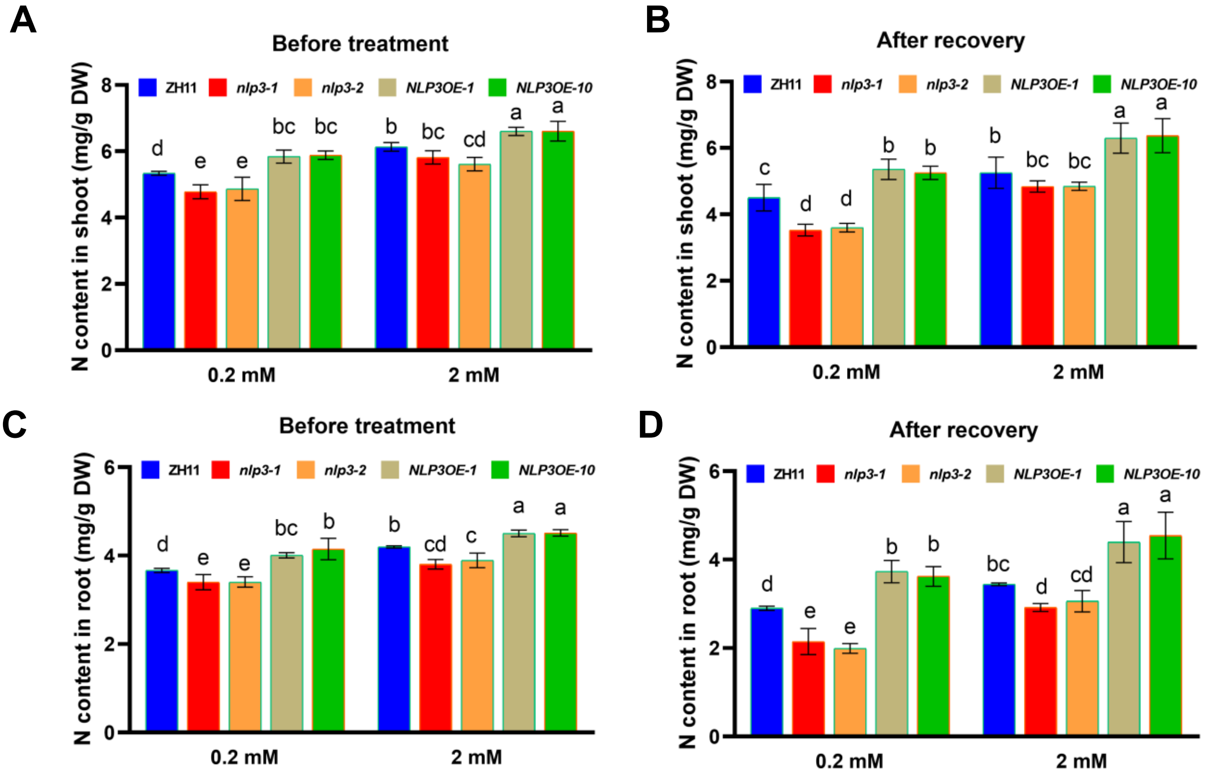

Figure S6. Nitrogen content in *nlp3* mutants and *NLP3* overexpression plants. Wild-type (ZH11), *nlp3* mutant (*nlp3-1/-2*), and *NLP3* overexpression (*NLP3OE-1/-10*) plants grown under 0.2 mM and 2 mM nitrogen conditions at 29°C were subjected to heat stress (45°C) treatment for 3 d and then recovered at 29°C for 7 d. Shoots and roots collected before stress treatment or after 7 d recovery were grounded into fine powder to measure total nitrogen content. Error bars represent SE (n = 3). Different letters indicate significant differences as determined by a Tukey's HSD test ($P < 0.05$).

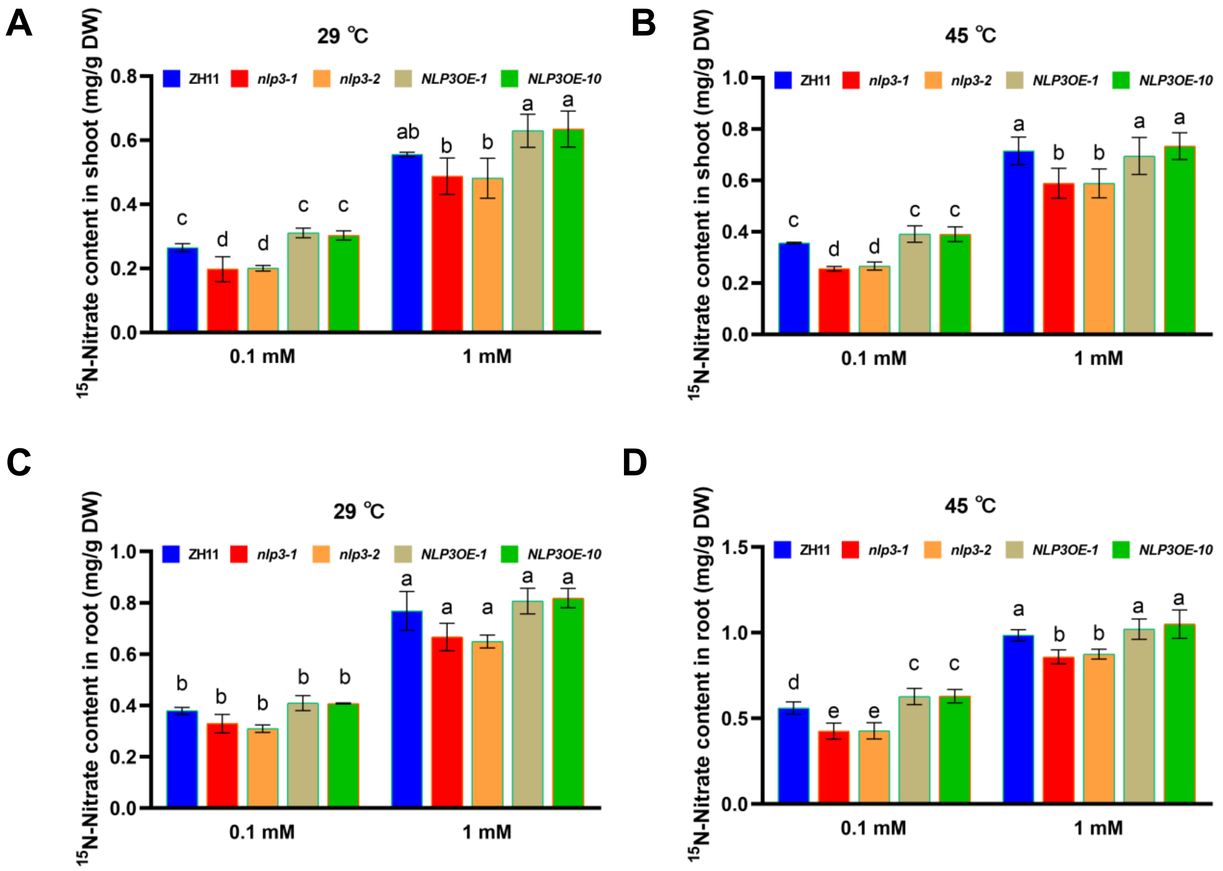

Figure S7. Nitrogen uptake assay with ^{15}N-nitrate. Seven-day old wild-type (ZH11), *nlp3* mutant (*nlp3-1/-2*), and *NLP3* overexpression (*NLP3OE-1/-10*) plants grown under 0.1 mM and 1 mM ^{15}N-KNO3 solution were transferred from 29℃ to 45℃ (heat stress) for 2 d. Shoots and roots were collected to measure ^{15}N-nitrate content. Error bars represent SE (n = 3). Different letters indicate significant differences as determined by a Tukey's HSD test ($P < 0.05$).

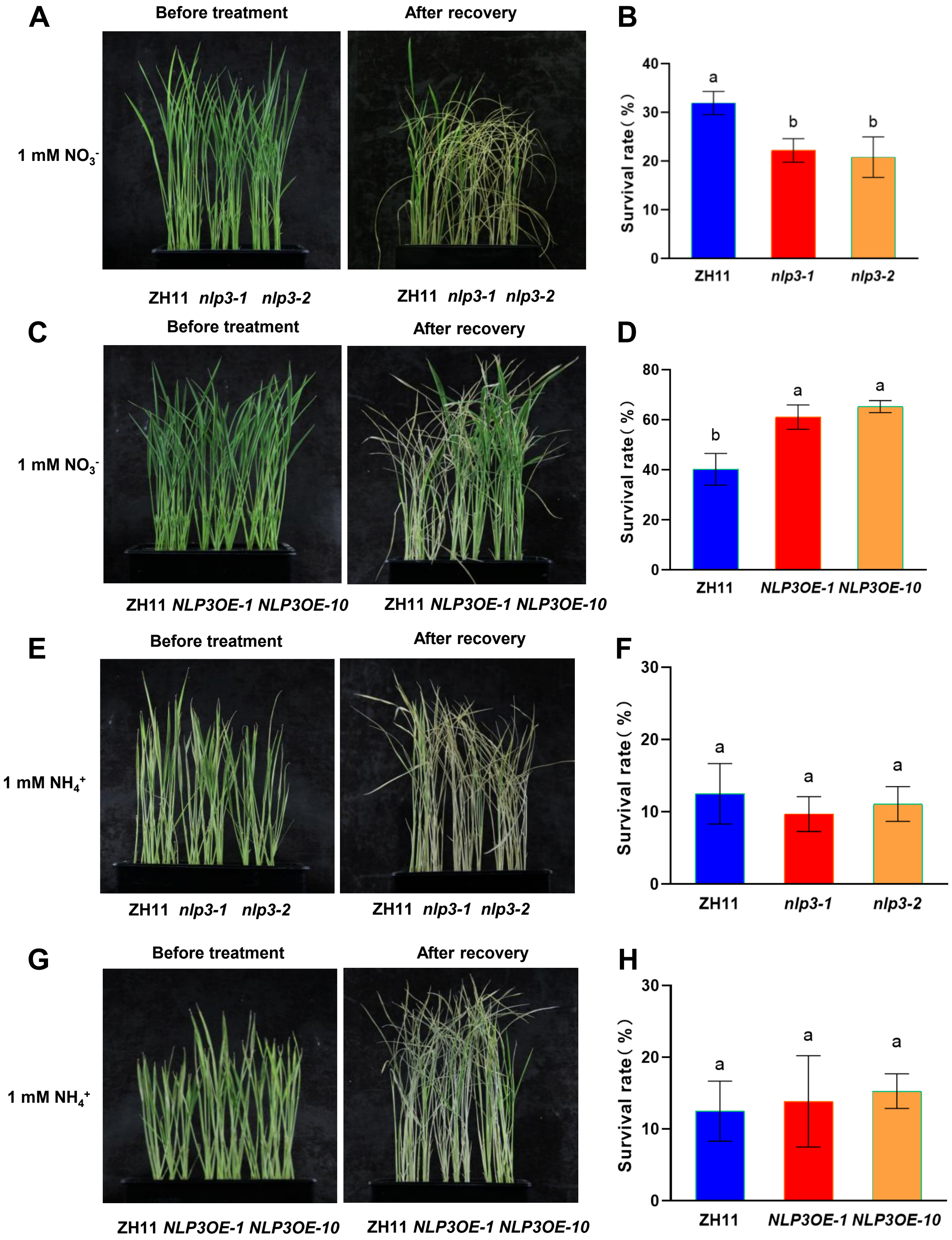

Figure S8. Phenotypic analysis under pure nitrate or pure ammonium conditions. Wild-type (ZH11), *nlp3* mutant (*nlp3-1* and *nlp3-2*), and *NLP3* overexpression (*NLP3OE-1* and *NLP3-10*) plants grown under pure nitrate (A-D) or pure ammonium (E-H) conditions at 29°C were subjected to heat stress (45°C) treatment for 3 d and then recovered at 29°C for 7 d, and plants were photographed and survival rate was calculated. Error bars represent SE (n = 3). Different letters indicate significant differences as determined by a Tukey's HSD test ($P < 0.05$).

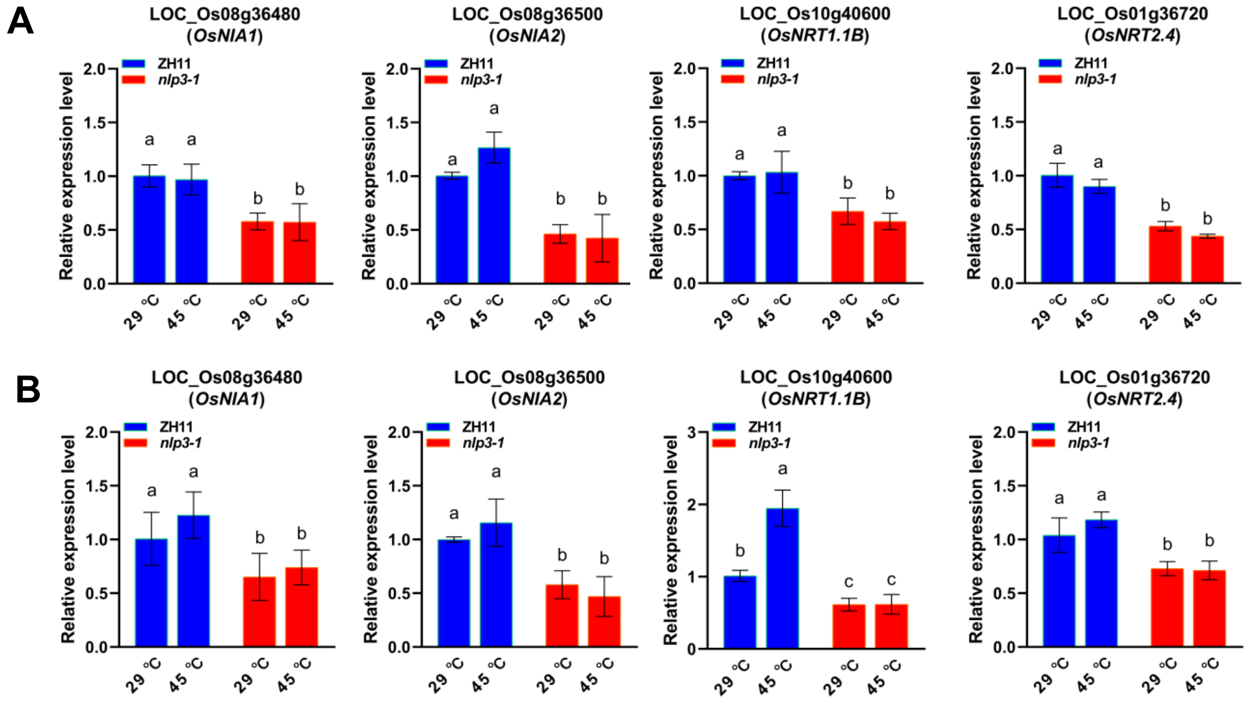

Figure S9. Gene expression analysis of nitrogen metabolism genes. Plants grown under 0.2 mM (A) and 2 mM (B) mixed nitrogen conditions at 29°C were subjected to 45°C treatment for 2 hr and harvested for RT-qPCR analysis. Error bars represent SE (n = 3). Different letters indicate significant differences as determined by a Tukey's HSD test ($P < 0.05$).

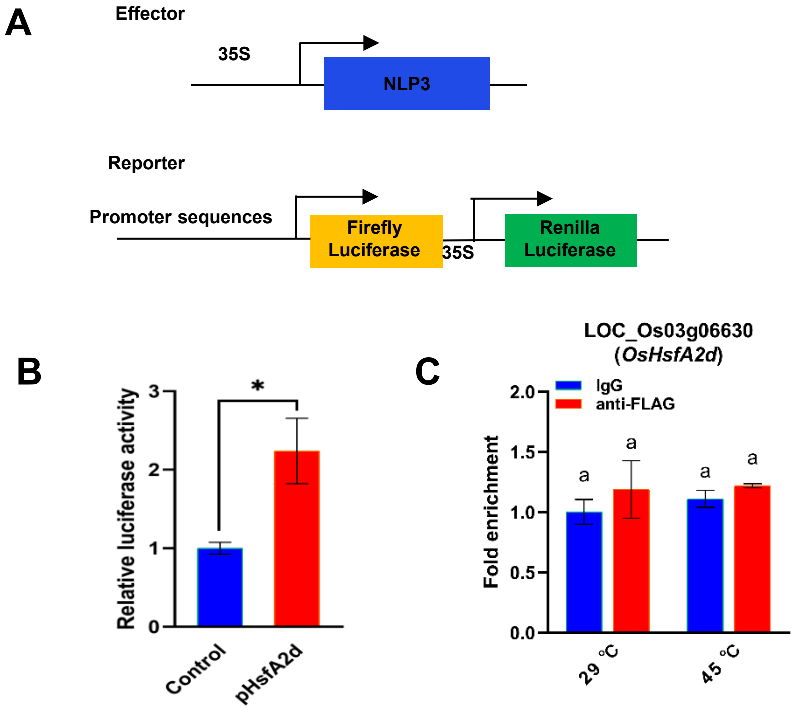

Figure S10. NLP3 does not directly control the expression of HsfA2d. *HsfA3/A7/A2d* promoter sequence or NRE-like motif (TTGACC) is linked with firefly luciferase to make the reporter while constitutively expressed NLP3 is an effector, in which Renilla luciferase driven by 35S promoter is used as an internal control (A). The relative luciferase activity is the firefly luciferase activity normalized to the Renilla luciferase activity which was then normalized to the empty vector control (B). 14-day-old *NLP3-FLAG* overexpression plants grown at 30ºC under N normal conditions were subjected to heat stress (45ºC) for 2 hr, and then harvested for ChIP-qPCR (C). Error bars represent SE (n = 3). Asterisks indicate significance levels when comparing to the control in *t*-test. (*, $P < 0.05$). Different letters indicate significant differences as determined by a Tukey's HSD test ($P < 0.05$).

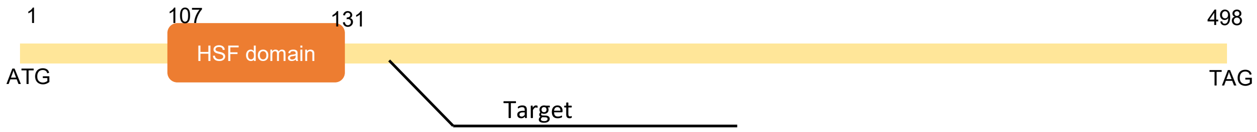


```
ZH11 (WT)    5′-CATGAGGATTTCCTACGACATAGCAAACATTTGTTGAAGAAGATTGTCCGG-CGCAGGTCCTCACCAACACAACAAAGTGGTCTTCAGCCTGGCTCT-3′
hsfa3-1 (+T) 5′-CATGAGGATTTCCTACGACATAGCAAACATTTGTTGAAGAAGATTGTCCGGTCGCAGGTCCTCACCAACACAACAAAGTGGTCTTCAGCCTGGCTCT-3′
hsfa3-2 (+G) 5′-CATGAGGATTTCCTACGACATAGCAAACATTTGTTGAAGAAGATTGTCCGGGCGCAGGTCCTCACCAACACAACAAAGTGGTCTTCAGCCTGGCTCT-3′
```

```
                 10        20        30        40        50        60        70        80        90       100
         ....|....|....|....|....|....|....|....|....|....|....|....|....|....|....|....|....|....|....|....|
ZH1      MDHNTDPPPTTMVDAAAALLLEPKLEGYDDDGGGEPLQPAPFVSPLDQLMQPPRPLEALLQGPQLPPFLSKTYDLVCEPELDGVISWGHAGNSFVVWDPS
hsfa3-1  MDHNTDPPPTTMVDAAAALLLEPKLEGYDDDGGGEPLQPAPFVSPLDQLMQPPRPLEALLQGPQLPPFLSKTYDLVCEPELDGVISWGHAGNSFVVWDPS
hsfa3-2  MDHNTDPPPTTMVDAAAALLLEPKLEGYDDDGGGEPLQPAPFVSPLDQLMQPPRPLEALLQGPQLPPFLSKTYDLVCEPELDGVISWGHAGNSFVVWDPS

                110       120       130       140       150       160       170       180       190       200
         ....|....|....|....|....|....|....|....|....|....|....|....|....|....|....|....|....|....|....|....|
Zh11     AFARDVLPHHFKHNNFSSFVRQLNTYGFRKVHADRWEFAHEDFLRHSKHLLKKIVRRRSSPTQQSGLQPGSSGESGLDPELNTLRREKSALLQEVTRLKQ
hsfa3-1  AFARDVLPHHFKHNNFSSFVRQLNTYGFRKVHADRWEFAHEDFLRHSKHLLKKIVRSQVLTNTTKWSSAWLFW*
hsfa3-2  AFARDVLPHHFKHNNFSSFVRQLNTYGFRKVHADRWEFAHEDFLRHSKHLLKKIVRAQVLTNTTKWSSAWLFW*

                210       220       230       240       250       260       270       280       290       300
         ....|....|....|....|....|....|....|....|....|....|....|....|....|....|....|....|....|....|....|....|
ZH11     EHLQTIEQMSTLNQRLESAEDRQKQMVSFLAKLLQNPTFLRQLKMHRQQKEIDSTRVKRKFLKHVPHGNIDSGESSSQHTGESNLDFSPTSLDLPATHSD
hsfa3-1
hsfa3-2

                310       320       330       340       350       360       370       380       390       400
         ....|....|....|....|....|....|....|....|....|....|....|....|....|....|....|....|....|....|....|....|
ZH11     ILDLQNFLLEDGDLNLAMLPENIGLDGIEAPDDIGALVQGFDTQEELELGSGVELLEIPPASGPRGQDPTIGRSKGKNVLSPGLDATSSEADCLGSFSDN
hsfa3-1
hsfa3-2

                410       420       430       440       450       460       470       480       490       500
         ....|....|....|....|....|....|....|....|....|....|....|....|....|....|....|....|....|....|....|....|
ZH11     MGMLSDSMLQTAGKLMDADDDERIWGVDASSALQSSCSGTSQQAYGSLVSDPYLMEMANKPEKFWELDFQALDDGDLQLDKCVIDDPALQQQRGNMNS*
hsfa3-1
hsfa3-2
```

Figure S11. Characterization of *hsfa3* mutants. The mutated nucleic acids and protein sequences of HsfA3 in wild-type ZH11 and gene-edited mutants (*hsfa3-1/-2*).

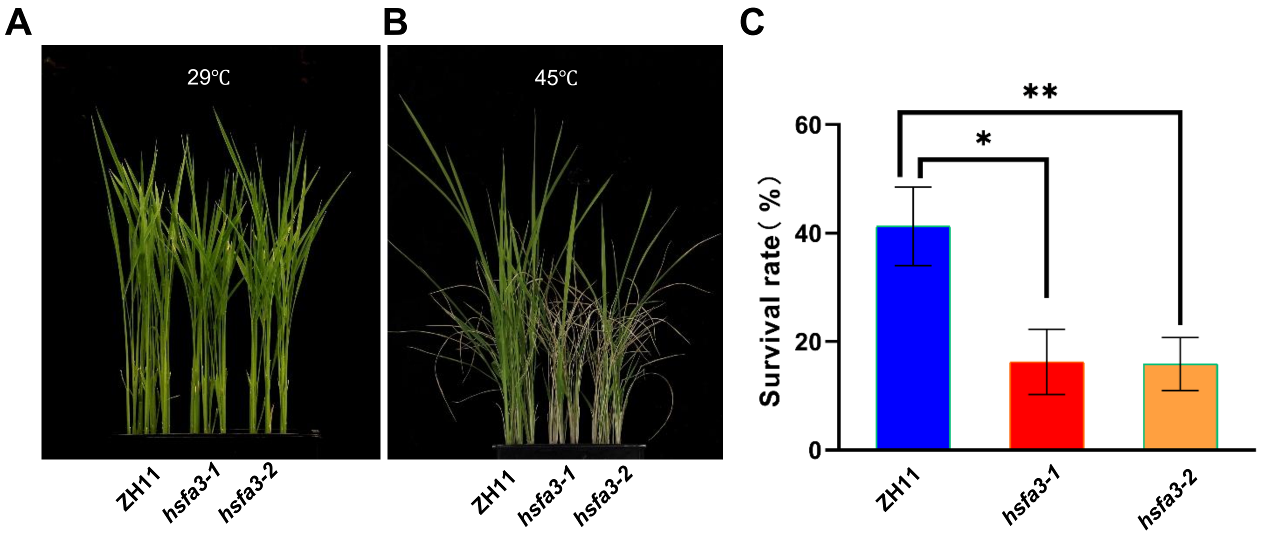

Figure S12. Phenotypic analysis of the *hsfa3* mutants. Seven-day-old wild-type (ZH11), mutants of *HsfA3* (*hsfa3-1/hsfa3-2*) grown at 29°C were subjected to heat stress (45°C) for 2 d and then recovered at 29°C for 14 d, and plants were photographed (A-B) and survival rate was calculated (C). Error bars represent SE (n=3). Asterisks indicate significance levels when comparing to ZH11 in *t*-test (*, P<0.05; **, P<0.01).
